# The atypical ‘hippocampal’ glutamate receptor coupled to phospholipase D that controls stretch‐sensitivity in primary mechanosensory nerve endings is homomeric purely metabotropic GluK2

**DOI:** 10.1113/EP090761

**Published:** 2023-09-01

**Authors:** Karen J. Thompson, Sonia Watson, Chiara Zanato, Sergio Dall'Angelo, Joriene C. De Nooij, Bethany Pace‐Bonello, Fiona C. Shenton, Helen E. Sanger, Beverly A. Heinz, Lisa M. Broad, Noelle Grosjean, Jessica R. McQuillian, Marina Dubini, Susan Pyner, Iain Greig, Matteo Zanda, David Bleakman, Robert W. Banks, Guy S. Bewick

**Affiliations:** ^1^ Institute of Medical Sciences, School of Medicine, Medical Sciences & Nutrition University of Aberdeen Aberdeen UK; ^2^ Department of Neurology Columbia University New York NY USA; ^3^ Department of Biosciences Durham University Durham UK; ^4^ Eli Lilly Bracknell UK; ^5^ CNRS UMR 5297, Interdisciplinary Institute of Neuroscience University of Bordeaux Bordeaux France

**Keywords:** GluK2, glutamate receptor, kainate receptor, mechanosensation, muscle spindle, PLD‐mGluR

## Abstract

A metabotropic glutamate receptor coupled to phospholipase D (PLD‐mGluR) was discovered in the hippocampus over three decades ago. Its pharmacology and direct linkage to PLD activation are well established and indicate it is a highly atypical glutamate receptor. A receptor with the same pharmacology is present in spindle primary sensory terminals where its blockade can totally abolish, and its activation can double, the normal stretch‐evoked firing. We report here the first identification of this PLD‐mGluR protein, by capitalizing on its expression in primary mechanosensory terminals, developing an enriched source, pharmacological profiling to identify an optimal ligand, and then functionalizing it as a molecular tool. Evidence from immunofluorescence, western and far‐western blotting indicates PLD‐mGluR is homomeric GluK2, since GluK2 is the only glutamate receptor protein/receptor subunit present in spindle mechanosensory terminals. Its expression was also found in the lanceolate palisade ending of hair follicle, also known to contain the PLD‐mGluR. Finally, in a mouse model with ionotropic function ablated in the GluK2 subunit, spindle glutamatergic responses were still present, confirming it acts purely metabotropically. We conclude the PLD‐mGluR is a homomeric GluK2 kainate receptor signalling purely metabotropically and it is common to other, perhaps all, primary mechanosensory endings.

## Abstract (highlights)

- **What is the central question of this study?**
  The metabotropic glutamate receptor coupled to phospholipase D (PLD‐mGluR) is a glutamate receptor previously only characterized pharmacologically but essential for maintaining stretch responsiveness in muscle spindle mechanosensory primary endings: what is the PLD‐mGluR protein?
- **What is the main finding and its importance?**
  PLD‐mGluR was identified as a homomeric GluK2 receptor signalling metabotropically. This identifies PLD‐mGluR 30 years after its discovery. This is important because: PLD‐mGluR is essential for muscle spindle stretch sensitivity; it is the first native kainate receptor shown to signal solely metabotropically; and, as it is the only GluR expressed in spindle mechanosensory endings, muscle spindles make a good functional assay of the native receptor.

## 1 INTRODUCTION

Mechanosensitivity is essential for life, from the regulation of cell volume and limb positioning, through maintaining adequate blood pressure for brain perfusion, to sensing the external environment. Consequently, tight control of the output from detectors of mechanical stimuli is vital.

A hitherto little‐known mechanism for regulating sensory output from mechanosensory nerve terminals is an intrinsic modulation whereby classical neurotransmitters are released by the sensory terminals when stretched, to act autogenically directly on the same terminals to fine‐tune the ending sensitivity. This was first described in the oval organ of the lobster scaphognathite, which releases the peptide proctolin (Pasztor & Bush, 1987; Pasztor et al., 1988). We demonstrated a similar mechanism in the mammalian muscle spindle (Banks et al., 2002; Bewick et al., 2005) and lanceolate endings of hair follicles (Banks et al., 2013), while others also reported it in visceral mechanosensory afferents (Blackshaw, 2001; DeGroot et al., 2000; Page et al., 2005). This suggests such systems are ubiquitous in mechanosensory endings. In mammalian endings, the commonest modulator is the amino acid glutamate which, in the systems we have studied, is released from vesicles resembling those in presynaptic terminals, to autogenically enhance mechanosensory responsiveness (Bewick, 2015; Bewick & Banks, 2015, 2021; Bewick et al., 2005; Than et al., 2021). In visceral vagal afferents, glutamate inhibits mechanosensitivity via classical metabotropic glutamate receptors (mGluRs 4, 6, 7 and 8) and excites via kynurenate‐sensitive ionotropic receptors (Page et al., 2005). The latter are commonly thought to be NMDA receptors in other peripheral afferents (Cahusac et al., 2005; Gazerani et al., 2010). GABAergic systems have also been reported (Blackshaw, 2001). However, neither GABA nor kynurenate sensitivity was found in the muscle spindle modulatory system (Bewick et al., 2005). In the present study, therefore, we seek to identify the glutamate receptor in muscle spindle primary afferents. The principal known characteristics of the system in these mammalian mechanosensory endings are:

- high expression of glutamate and vesicular glutamate transporters (vGluT1);
- populations of numerous small, clear ('synaptic‐like') vesicles (SLVs);
- Ca^2+^‐dependent SLV recycling locally, constitutively at rest, which is enhanced by mechanical activity;
- glutamate increases SLV recycling and/or stretch‐evoked action potential output, via a glutamate autoreceptor which is blocked by the specific antagonist PCCG‐13;
- manipulations preventing receptor activation (receptor antagonism, blockade of SLV exocytosis or Ca^2+^ channels) can silence the sensory ending.

The receptor protein in muscle spindle and hair follicle lanceolate afferent terminals is clearly not a classical, characterised glutamate receptor. Its actions are unaffected by kynurenate, the broad‐spectrum inhibitor of the ionotropic glutamate receptors (NMDA, AMPA and kainate iGluRs), or inhibitors of group I–III mGluRs (Banks et al., 2013; Bewick et al., 2005). However, the excitation by exogenous glutamate is profoundly *inhibited* by the classical *agonist* of group I mGluRs, (*RS*)‐3,5‐dihydroxyphenylglycine (DHPG) and by (2*R*,1′*S*,2′*R*,3′*S*)‐2‐(2′‐carboxy‐3′‐phenylcyclopropyl) glycine (PCCG‐13). These findings most closely align with the properties of the non‐canonical GluR first identified in the hippocampus, directly coupled to phospholipase D (PLD). This has been termed the metabotropic glutamate receptor coupled to phospholipase D (PLD‐mGluR), for which PCCG‐13 is the selective antagonist (Albani‐Torregrossa et al., 1999; Pellegrini‐Giampietro et al., 1996). Thus, the only glutamate receptor detectable pharmacologically in these primary mechanosensory terminals is the PLD‐mGluR. Here it acts like a sensory ‘volume control’, powerfully regulating output between a total silencing and a doubling of the stretch‐evoked output. Indeed, prolonged exposure (1–6 h) to PCCG‐13 completely blocks both the electrical output of spindles and SLV endocytosis in hair follicle lanceolate afferents (Banks et al., 2013; Bewick et al., 2005).

Given its pivotal importance and unusual pharmacology, the present study used the muscle spindle as an assay tissue to identify this atypical ‘PLD‐mGluR’ protein. Spindle primary mechanosensory terminals facilitated PLD‐mGluR isolation due to their large size, their position at a long separation from the neuronal soma and synaptic endings, which enabled ready differentiation of peripheral expression. Screening was further aided by using a tissue highly enriched for muscle spindles and further extending the existing pharmacological screening to identify a good affinity probe for target cross‐validation with an immunolabelling screen. Ultimately, we found only a single glutamate receptor subunit protein expressed, which was validated physiologically and pharmacologically in a mouse model lacking ionotropic function of the putative receptor. The expression of the same protein was then shown in hair follicle lanceolate endings. In summary, the only glutamate receptor protein detectable in spindle mechanosensory terminals was kainate receptor subunit 2 (GluK2), which was also then found in hair follicle lanceolate mechanosensory terminals. We conclude, therefore, that the PLD‐mGluR in primary mechanosensory nerve terminals is GluK2, whose atypical pharmacology reflects its unusual homomeric expression, independent of other glutamate receptor proteins.

This identification of PLD‐mGluR as homomeric GluK2 and as the only GluR expressed, means muscle spindles are ideal for functional studies of this unusual receptor in its native form in an intact system.

## 2 METHODS

For full methodological details, see Supporting information (S1–S9).

### 2.1 Animals

All animal work was in accordance with the UK Animals (Scientific Procedures) Act 1986, Amendment Regulations, 2012, and approved locally by the University of Aberdeen Animal Welfare and Ethical Review Board. Rats (adult Sprague–Dawley, either sex and >250 g) and mice (wild type and GluK2‐Neo, with a Neo cassette disrupting ionotropic function) were used in the studies. Unless stated otherwise, animals were killed by approved Schedule 1 methods (rats: steadily increasing CO_2_ levels to unconsciousness; mice: cervical dislocation; both methods were followed by decapitation) before tissues were removed (Supporting information S1).

### 2.2 Electrophysiology

Electrophysiological recordings of stretch‐evoked spindle responses from rat lumbrical and mouse soleus muscles (Bewick et al., 2005; Lin et al., 2016) were as described previously. Briefly, nerve–muscle preparations were dissected and placed in gassed (95% O_2_–5% CO_2_) bicarbonate‐buffered physiological saline at room temperature (RT; 17–20°C), and then the evoked electroneurogram was recorded by laying the nerve across silver wire electrodes (rat lumbrical) or taking it into a tight‐fitting glass suction electrode (mouse soleus). Amplified signals were processed and analysed using Spike2 software (Cambridge Electronic Design, Cambridge, UK) (Supporting information S2).

### 2.3 Deep masseter dissection, cryo‐sectioning, spindle column localization and 3D reconstruction

The rat deep masseter muscle contains a continuous column of densely packed muscle spindles. For the column's full characterization (location, composition, histological examination and 3‐dimensional (3D) image reconstruction), the rostral half of the muscle, containing the muscle spindle column, was sectioned and sections stained with haematoxylin and eosin. The sections were viewed with a modified Nikon Optiphot 2 upright microscope (MicroInstruments, Oxford, UK). Images were captured using a Retiga EXi camera and Volocity software (Perkin‐Elmer, Beaconsfield, UK). 3D modelling used Reconstruct software2 (NIH Software; https://www.bu.edu/neural/Reconstruct.html) whereby individual muscle spindle transected profiles were traced manually in each section and compiled across all sections to locate each of them within the entire tissue (Supporting information S3).

### 2.4 Enriched muscle spindle homogenate preparation

For molecular and biochemical investigation of the PLD‐mGluR, the deep masseter spindle column was dissected as an enriched source of spindle terminal protein. Briefly, after gentle collagenase digestion (0.1% Type I collagenase, 37°C) to dissociate the muscle spindle column from the surrounding muscle, the tissue was stained with methylene blue (0.1%) to visualize the muscle spindles for isolation by microdissection. Isolated spindle columns and spindle‐free rostro‐lateral muscle portions (control) were then homogenized and probed by molecular and biochemical methods (Supporting information S4).

### 2.5 Novel PLD‐mGluR agonist ligand analogues as screening tools

Chemical structures of active ligands from our pharmacological screens of PLD‐mGluR were assessed for suitability for modification to accept tags as affinity probes. The specific antagonist PCCG‐13 was discounted as both intrinsically difficult to synthesize and modification would likely destroy its precise 3D structure. Kainate was therefore selected as both a highly effective agonist and readily amenable to chemical functionalization to the 4‐(1,2,3‐triazolyl) analogue (ZCZ90) (Zanato et al., 2014). ZCZ90, retained similar agonist potency to kainate itself, which was blocked by the selective PLD‐mGluR antagonist PCCG‐13 (Zanato et al., 2014). Addition of biotin or fluorescein via a *O*‐(2‐aminoethyl)‐*O′*‐(2‐azidoethyl) pentaethylene glycol polymeric chain generated the compounds ZCZ180 (Zanato et al., 2014) and ZCZ172 (Supporting information S10) for further screening. ZCZ180 was used for far‐western affinity blotting and ZCZ172 for fluorescence affinity labelling of spindle primary sensory terminals in whole‐mount preparations for microscopic observation.

### 2.6 Isolation of (*R*)‐3,5‐DHPG from (*RS*)‐3,5‐DHPG

(*R*)‐DHPG was obtained from (*RS*)‐3,5‐DHPG (Supporting information S11) by chiral HPLC resolution of a commercial racemic mixture (Bio‐Techne, Abingdon, UK) using an optimized method derived from a published procedure (Garcia & Azerad, 1997). The desired enantiomer was eluted using a Daicel Crownpack CR (−) column (4 × 150 mm, 5 μm) (Daicel Chiral Technologies, West Chester, PA, USA), and isocratic elution using HClO_4_ aqueous solution at pH 1.5, flow rate of 0.6 ml/min, 15°C. The peak related to (*R*)‐DHPG was identified by comparison of peaks derived from injection of the racemic mixture and injection of an (*S*)‐DHPG standard (run time (Rt) of (*S*)‐DHPG (Tocris) = 5.9 min, Supporting information Figure S11.2a; Rt of (*R*)‐DHPG = 8.5 min, Supporting information Figure S2.2b). The fractions containing (*R*)‐DHPG were combined and lyophilized giving a brown oil. Residual perchloric acid and perchlorate counter ions were removed using an ion‐exchange column (Strata‐X‐C, Phenomenex, Macclesfield, UK; 33 μm, 200 mg/3 ml; protocol reported in Supporting information S11). (*R*)‐DHPG was obtained with a purity higher than 98% (Supporting information Figure S11.2c).

### 2.7 Ligand FLIPR screening assays against human mGluRs

#### 2.7.1 Compounds

l‐Cysteinesulfinic acid monohydrate (l‐CSA; Merck Life Science, Gillingham, UK), (2*R*,4*R*)‐APDC, (*RS*)‐baclofen, MPEP and NS3763 (Tocris), LY341495 (Eli Lilly, Bracknel, UK), PCCG‐13 (gift from Prof. Roberto Pellicciari and Prof. Maura Marinozzi, University of Perugia, Italy), ZCZ compounds (Zanda laboratory).

#### 2.7.2 Animals and cell culture

Rat cortical neurones were prepared from the excised brains of embryonic day 18 Sprague–Dawley rats (Harlan, Oxford, UK). All animals were treated in accordance with the UK Animal Scientific Procedures Act 1986, and all procedures were approved through the UK Home Office Inspectorate. Pregnant rats were first anaesthetized in a rising concentration of CO_2_ atmosphere then killed by cervical dislocation. Embryos were removed under sterile conditions. The neocortices were prepared (Brewer, 1995) and 100 μL of dissociated neurones were plated into 96‐well poly‐d‐lysine‐coated plates (Biocoat, BD Biosciences, Wokingham, UK) at a cell density of 60 × 104 cells per ml then incubated at 37°C for 9 days prior to the experiment.

#### 2.7.3 Measurement of [Ca^2+^]_i_ using FLIPR

Medium was removed and cells were incubated in HEPES‐buffered Tyrode's solution containing Ca^2+^ and Mg^2+^ (Thermo Fisher Scientific, Inchinnan, UK) and 4 μM Fluo 3‐AM/0.05% pluronic F127 (Thermo Fisher Scientific) for 1 h in the dark. The solution was then replaced with HEPES‐buffered Tyrode's solution minus Ca^2+^ and Mg^2+^ plus 2.5 mM CaCl_2_ (= Mg^2+^‐free buffer) ± inhibitor cocktail (10 μM NS3763, 10 μM LY341495 and 1 μM MPEP(2‐methyl‐6(phenylethynyl)pyridine) immediately before transferring the plate onto the FLIPR (Molecular Devices, Wokingham, UK) assay system. Compound addition was automated and added after a baseline read of 300 s. Fluorescence signals were recorded at 1 s intervals with a 0.4 s exposure time.

### 2.8 Western blotting

Deep masseter spindle column isolates from 10 rats (20 muscles) were pooled, giving ∼2000 primary nerve endings per sample. The positive control was from hippocampus, except for mGluR6 (not expressed in hippocampus), for which retina was used. The negative control was lateral deep masseter, which is largely devoid of spindles.

Spindle isolates were homogenized in 6× Laemmli buffer (56 mM Tris–HCl pH 6.8, 2.1% SDS, 11.2% glycerol, 11 mM dl‐dithithreitol plus bromophenol blue) and loaded onto a stacking gel above an 8% polyacrylamide separation gel, plus a lane of molecular mass marker (Amersham, GE Healthcare Life Sciences, Chalfont St Giles, UK). Samples were separated (SDS‐PAGE, 150 V, 75 min) in Tris–glycine–SDS running buffer (14.9 mM Tris; 95.9 mM glycine; 1.7 mM SDS) and proteins transferred onto nitrocellulose membrane (150 V, 90 min) in Tris–glycine–methanol transfer buffer (24.8 mM Tris, 192 mM glycine, 200 mM methanol). Protein transfer was confirmed by Ponceau staining, which was removed (3× washes in distilled H_2_O).

Non‐specific antibody binding was blocked with 5% milk + Tris‐buffered saline (TBS; 19.8 mM Tris base; 150 mM NaCl; pH 7.6) with 0.05% Tween (TBST) (1 h, RT). Membranes were then incubated in primary antibody diluted in 5% milk + TBST and either incubated overnight at 4°C, or for 1 h at room temperature (for antibody details see Supporting information Table S2).

Unbound primary antibody was removed in TBST (3 × 5 min) and then membranes were incubated in horseradish peroxidase (HRP)‐conjugated secondary antibody (5% milk + TBST, RT, 1 h; for antibody details see Supporting information Table S3).

### 2.9 Far‐western (affinity) blotting of gels

Proteins were separated by SDS‐PAGE and transferred to nitrocellulose membrane as above. Non‐specific binding was blocked with 5% bovine serum albumin (BSA; Sigma‐Aldrich) in TBST (RT, 1 h) and membranes incubated in 100 μM ZCZ‐180 diluted in 5% BSA with TBST (4°C, overnight). Membranes were washed (3 × 5 min, TBST) then incubated in HRP‐conjugated streptavidin (RT, 1 h in 1:2000; Thermo Fisher Scientific). Labelling was detected using a Pierce Enhanced Chemiluminescence Substrate kit (Thermo Fisher Scientific) according to the manufacturer's protocol. They were then either exposed onto X‐ray film and developed (Kodak X‐Omat 1000 Processor, Eastman Kodak, Rochester, NY, USA), or imaged at 302 nm excitation (FluorChem FC2 MultiImage II imager), and images captured using AlphaView software (Alpha Innotech, San Leandro, CA, USA).

### 2.10 Immunolabelling and affinity labelling with ZCZ172 in whole‐mounts

#### 2.10.1 Spindles

Rat lumbrical muscles were fixed (4% formaldehyde in phosphate‐buffered saline (PBS) (Millipore, Livingston, UK), 4°C overnight), rinsed (3× PBS), cleaned of adherent tissue then teased. Muscles were squashed firmly between microscope slides to burst the spindle capsules, then pinned in the PDMS‐lined (polydimethylsiloxane; Sylgard 184, Dow Corning, Cheadle, UK) Petri dish. Preparations were blocked (1% BSA, 0.4% Triton X‐100 in PBS, 1 h, RT) and incubated in primary antibodies (Supporting information Table S2) in PBS with 1% BSA and 0.1% Triton X‐100 (48 h, 4°C). Preparations were then washed (3 × 5 min, PBS) and incubated in relevant secondary antibody (Supporting information Table S3) in PBS with 1% BSA and 0.1% Triton X‐100 in the dark (1 h, RT). Where relevant, the fluorescein‐labelled affinity probe ZCZ‐172 was added (75 μM) to the secondary antibody diluent. For imaging, labelled preparations were again squashed between microscope slides to ease spindle visualization, then mounted (8% Mowiol 4‐88, Merck Life Science; 166 mM glycerol, 83 mM Tris pH 8.5, 223 mM 1,4‐diazabicyclo(2.2.2)octane (DABCO)) and coverslipped. Wide‐field images were captured using ×10 or ×25 objectives, with (red/green emission, respectively) excitation wavelengths of 450−490/510–560 nm. Confocal microscopy used a LSM710 inverted confocal microscope (Zeiss Microscopy, Jena, Germany) with laser excitation wavelengths of 488 (green) and 550 nm (red) and captured using Zen Blue 2012 software (Zeiss). Full details in Supporting information S6.

#### 2.10.2 Hair follicle afferents

Adult male Wistar rats were terminally anaesthetized with pentobarbital (Euthatal, Merial Animal Health Ltd, Harlow, UK) and upon cessation of respiration, perfused with heparinized saline followed by 4% paraformaldehyde. Inner ear skin was removed, post‐fixed overnight and then transferred to 30% sucrose in 0.1 M phosphate buffer (PB) pH 7.4 for a minimum of 24 h before freezing at −20°C in preparation for collecting 20 μm cryosections onto poly‐l‐lysine‐coated slides. Non‐specific binding sites were blocked with 10% NGS (normal goat serum; Abcam, Cambridge, UK) and 0.1% Triton‐X‐100 in PB for 40 min, rinsed in PB (1 × 10 min) then incubated in primary antibodies for GRIK2 and synaptophysin (Supporting information S8) in 1% NGS–0.1% Triton X‐100 in PB) overnight at 4°C. After washing (×3 in PB), the secondary antibodies (Supporting information S8) were applied for 2 h at RT. Following further washing (×3 in PB), the sections were mounted in Prolong Gold (Thermo Fisher Scientific) for viewing and image acquisition. Sections were examined using an Axioskop 2 (Zeiss) microscope under epifluorescence. Digital images were captured with an Orca 285 CCD camera (Hamamatsu Photonics, Welwyn Garden City, UK) controlled by Improvision Volocity software (v. 6.2.1, Perkin Elmer, Beaconsfield, UK).

### 2.11 Immunofluorescence of GluK2‐Neo mouse soleus spindles

Expression of GluK2 was investigated in GluK2‐Neo mice (gift from Prof. Christoph Mulle; Mulle et al., 1998). The soleus and splenius capitis muscles were removed from perfusion‐fixed (as per hair follicle afferents) GluK2‐Neo and wild type (WT) mice (*n* = 3 each) and stored (30% sucrose, 4°C). The muscles were embedded in OCT and 20 μm longitudinal cryosections placed on charged slides and stored at −80°C.

Sections were rinsed with PBS, then non‐specific binding sites blocked (10% NGS, 4% Triton X‐100 in PBS) for 30 min at room temperature. The sections were then incubated (1% NGS–0.1% Triton X‐100 in PBS) in primary antibodies (Vandenbeuch et al., 2010) for synaptophysin and either GRIK2 or GluR6/7 (Supporting information S9) for 48 h at 4°C. After washing in PBS (15 min) the secondary antibodies (Supporting information Table 2 S9) were applied (2 h, room temperature, in the dark). Sections were rinsed as before and mounted (Citifluor AF4; Agar Scientific, Stansted, UK) and stored in the dark at 4°C. Labelling was examined as described above. Captured images were imported into Photoshop Creative Cloud (v21.2.4; Adobe, San Jose, CA, USA) to create annotated figures.

### 2.12 Data analysis and statistics

#### 2.12.1 Electrophysiology of rat muscle spindles

Average stretch‐evoked firing frequency was calculated from the first 0.5 s of the hold phase of each of the three cycles of a manually applied stretch.

#### 2.12.2 Electrophysiology of mouse muscle spindles

Average stretch‐evoked firing frequency was calculated from the hold phase of each of the four cycles of a trapezoid stretch applied by a software‐controlled (WinWCP, Strathclyde Electrophysiology Software, University of Strathclyde, Glasgow, UK) electromagnetic puller.

#### 2.12.3 FLIPR assays

A Microsoft Excel template plotted the data and detected peaks in individual wells. Oscillation frequency change was determined from the Ca^2+^ peaks per 300 s time interval before and after compound addition to derive a normalized response. Data were plotted using a four‐parameter logistic curve fit model (GraphPad Prism, GraphPad Software, San Diego, CA, USA). Data from each culture preparation were averaged over three to six cell plates. Data represent mean values ± SEM from the replicate cell plates.

#### 2.12.4 Differences in fluorescence intensity between GluK2‐Neo and WT mice

Fluorescence intensity was quantified using ImageJ (NIH, Bethesda, MD, USA; http://rsbweb.nih.gov/ij/) as described previously (Simon et al., 2010). Briefly, the ratio of raw intensities of anti‐GRIK2 (N‐terminus) or anti‐GluR6/7 (C‐terminus) antibodies relative to anti‐synaptophysin immunofluorescence for defined regions of interest around the sensory terminals was calculated. This ratiometric method controlled for antibody accessibility variation between samples, enabling quantitative comparison of immunostaining intensities between N‐terminus and C‐terminus in WT and GluK‐Neo mice.

#### 2.12.5 Statistical analysis

Muscle‐spindle electrophysiology and fluorescence microscopy were analysed using the Real Statistics plug‐in for Excel (www.real‐statistics.com/). Individual details are given with the results and figure legends. FLIPR assay dose–response curves were compared by two‐way ANOVA using GraphPad Prism, applying Dunnett's multiple comparison correction where appropriate.

## 3 RESULTS

**FIGURE 1:**
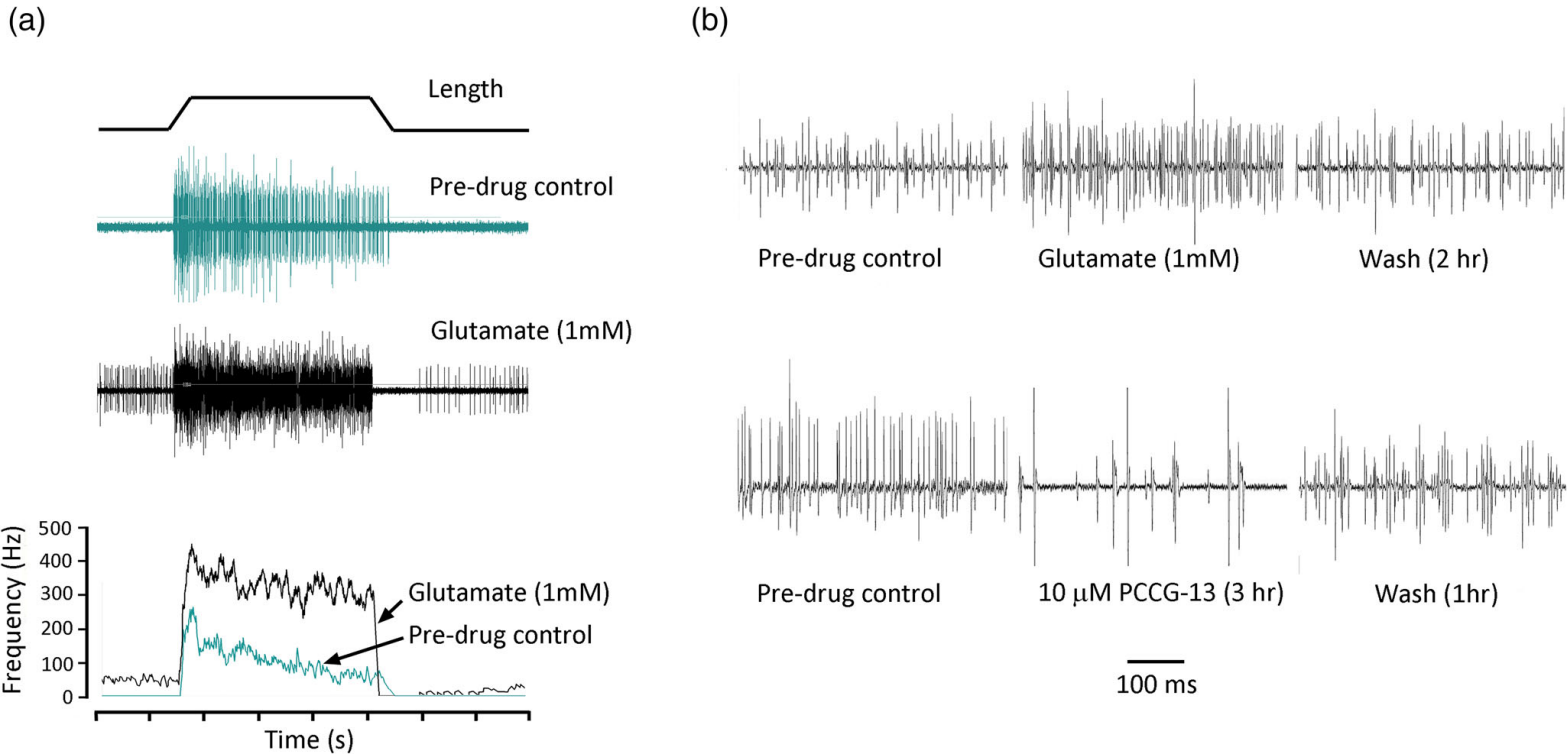
Whole nerve electroneurograms of stretch‐evoked responses from rat lumbrical muscles: firing enhanced by glutamate and inhibited by PCCG‐13. (a) Top: trapezoidal stretch‐hold‐release profile (1 mm stretch, ∼10% increase in length) used for all electrophysiology experiments. Middle: normal stretch response (cyan), and enhanced firing (black) in glutamate (1 mM, 1 h). Bottom: mean firing frequency profile for these responses, showing the glutamate‐mediated enhancement of firing (rolling average, sampled every 10 ms). (b) Initial 500 ms of hold phase firing, showing (top) glutamate (1 mM, 1 h) reversibly enhances and (bottom) PCCG‐13 (10 μM, 3 h) reversibly inhibits firing. These are illustrative repetitions of full experiments published earlier (Bewick et al., 2005). NB: action potential (AP) amplitude variation reflects the long duration of experiments (up to 14 h), reconnecting the nerve for each recording and AP summation in whole nerve recordings, so cannot be used to infer drug action.

Stretch‐evoked firing in spindles is enhanced by glutamate, an effect abolished by the specific PLD‐mGluR antagonist PCCG‐13 (Albani‐Torregrossa et al., 1999), while PCCG‐13 applied alone profoundly inhibits, even abolishes, firing (Figure 1 and Bewick et al., 2005). Thus, PLD‐mGluR is the only glutamate receptor involved. To help isolate this receptor, we first identified the optimal ligand to adapt as a molecular tool.

### 3.1 Pharmacological profiling of PLD‐mGluR to identify ligand suitable for functionalization

**FIGURE 2:**
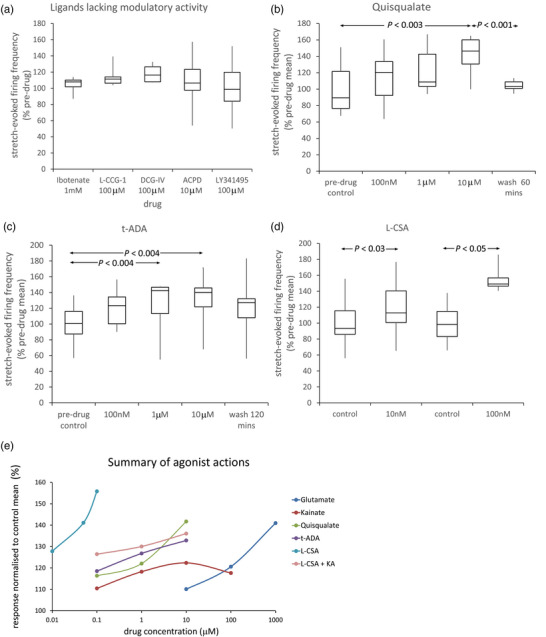
Further pharmacological characterization of PLD‐mGluR in muscle spindles. (a) Ligands without modulatory action included ibotenate (gp I mGluR agonist), l‐CCG‐I (gp II mGluR agonist), DCG‐IV (gp II mGluR and NMDA‐R agonist), ACPD (gp I, II and III mGluR agonist), and LY341495 (gp I, II and III mGluR antagonist at 100 μM). (b–d) In contrast, (b) quisqualate (AMPA, KAR and gp I mGluR agonist), (c) *t*‐ADA and (d) l‐CSA (weak gp I agonists) increase stretch‐evoked firing. (e) Partial dose–response relationships for effective agonists. Rank order of potency: l‐CSA > *t*‐ADA > quisqualate > kainate > glutamate. Kynurenic acid (KA, 1 mM) substantially inhibits the excitation by l‐CSA. (a–d) Individual data expressed relative to control grand mean; statistical analysis of raw data: one‐way ANOVA with replicates, post‐hoc pairwise paired Student's *t*‐test, significant differences shown after Benjamini–Hochberg correction method for multiple tests.

Common GluR ligands were tested for agonism or antagonism of stretch‐evoked firing (Figure 2, means from 4–10 muscles (m) from 3–10 rats (r) each). These were then compiled with those already tested in previous studies (Table 1).

**TABLE 1:** Summary GluR ligand pharmacological profile against spindle stretch‐induced firing.

	Known effect on canonical receptors							
	Metabotropic			Ionotropic			Effect on spindle firing (change and magnitude)	
Ligand (conc)	GpI	GpII	GpIII	NMDA	AMPA	KA	Alone	+ glutamate
No effect on spindle stretch‐evoked firing								
Kynurenate (1 mM)				–	–	–	×	= Glu alone
NBQX (10 μM)					–	–	×	= Glu alone
LY341495 (1 mM)	–	–	–				×	ND
MCPG (500 μM)	–	–					×	= Glu alone
CPPG (100 nM)		–					×	= Glu alone
MAP4 (1 mM)		–	–				×	= Glu alone
(*S*)‐3,5‐DHPG (200 μM)	+						×	= no Glu
Ibotenate (10 μM)	+						×	ND
ACPD (10 μM)	+	+					×	ND
l‐CCG‐I (10 μM)		+					×	ND
DCG‐IV (10 μM)		+		+			×	ND
l‐AP4 (10 μM)			+				×	ND
Active on spindle stretch‐evoked firing								
(*R*)‐3,5‐DHPG (100 μM)							– – –	ND
(*RS*)‐3,5‐DHPG (200 μM)	+						×	= no Glu
l‐CSA (10 nM)	+			+			+ + + +	ND
Quisqualate (10 μM)	+				+		+ + +	ND
*t*‐ADA (10 μM)	++						+ +	ND
Kainate (100 μM)						+	+ +	ND

*Note*: Ligand actions for canonical GluRs are indicated by font colour: red, antagonist; green, agonist; black, inactive. For PLD‐mGluR, — represents inhibition; **+** represents enhancement of firing; × represents no effect; ND, not done. Abbreviations: ACPD, 1‐aminocyclopentane‐*trans*‐1,3‐dicarboxylic acid (gp I/II agonist); *t*‐ADA, *trans*‐azetidine‐2,4‐dicarboxylic acid; CPPG, (*RS*)‐α‐cyclopropyl‐4‐phosphonophenylglycine (potent gp III antagonist); DCG‐IV (2*S*,1′*R*,2′*R*,3′*R*)‐2‐(2,3‐dicarboxycyclopropyl)glycine (gp II/III agonist); l‐AP4, l‐(+)‐2‐amino‐4‐phosphonobutyric acid (gp III agonist); l‐CCG‐I, (2*S*,1′*S*,2′*S*)‐l‐2‐(carboxycyclopropyl)glycine (gp II agonist); LY341495, (2*S*)‐2‐amino‐2‐[(1*S*,2*S*)‐2‐carboxycycloprop‐1‐yl]‐3‐(xantho‐9‐yl)propanoic acid (potent gpII antagonist); MAP4, (*S*)‐2‐amino‐2‐methyl‐4‐phosphonobutanoic acid (potent group II agonist, gp III antagonist); MCPG, (*RS*)‐α‐methyl‐4‐carboxyphenylglycine (gp I/II antagonist); NBQX, 2,3‐dihydroxy‐6‐nitro‐7‐sulfamoyl‐benzo[f]quinoxaline (AMPA/KA antagonist).

#### 3.1.1 Agonists

The agonist rank order (lowest dose significantly increasing firing) was l‐CSA > *t*‐ADA > quisqualate > kainate > glutamate (summarized in Figure 2e).

#### 3.1.2 Antagonists

**FIGURE 3:**
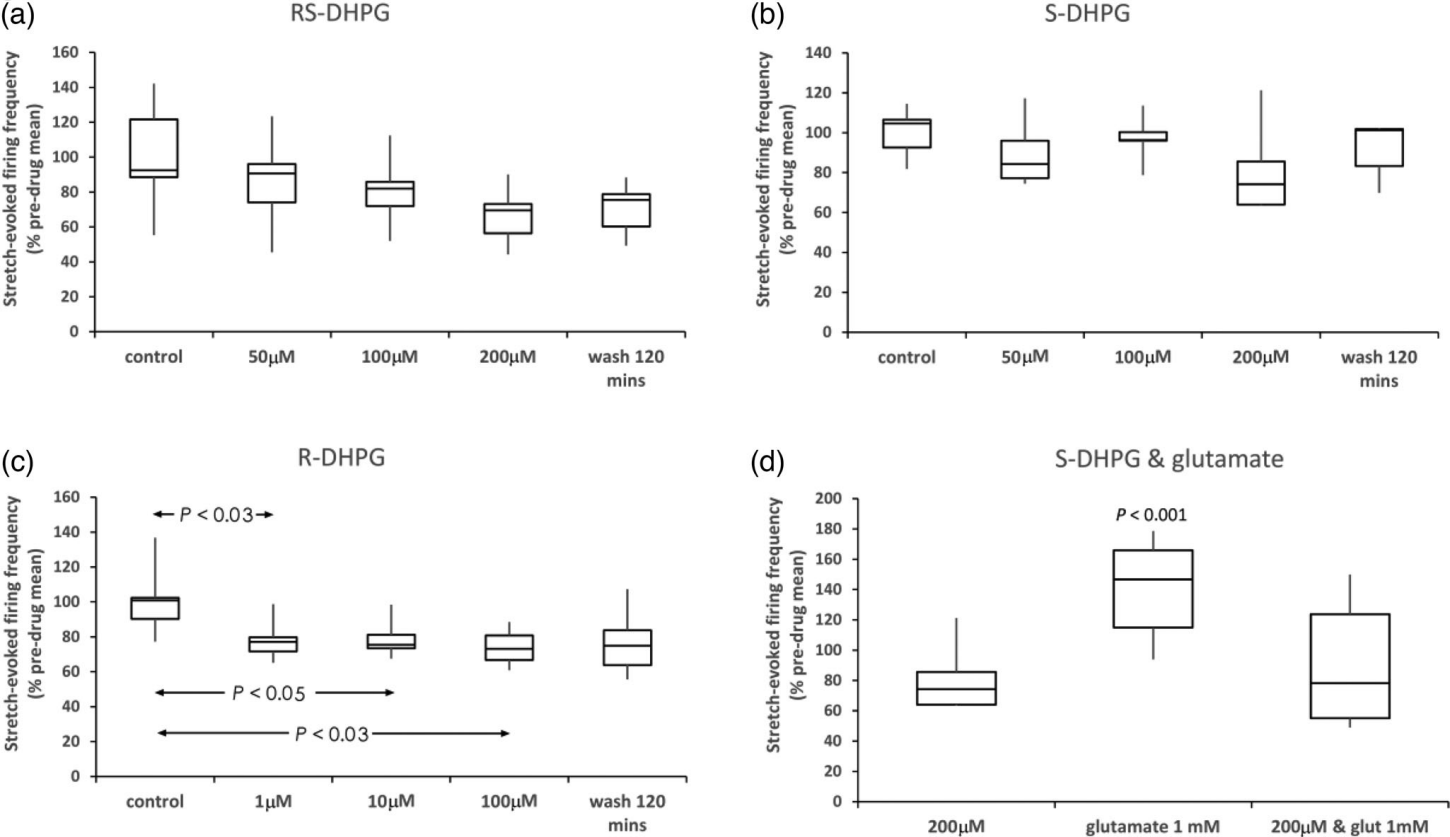
The ‘inert’ enantiomer (*R*)‐3,5‐DHPG inhibits the PLD‐mGluR. The racemic (*RS*)‐3,5‐DHPG mixture blocks enhancement of stretch‐evoked firing in spindles by exogenous glutamate (Bewick et al., 2005). (a) Applied alone, it tended to decrease firing, but did not reach significance (*m* = 5 muscles; r = 5 rats). (b) (*S*)‐DHPG had no effect on firing up to 200 μM (*m* = 5, r = 5). (c) (*R*)‐DHPG (inactive enantiomer against gp I‐III mGluRs) significantly decreased it even at 1 μM (*m* = 7, r = 5), reversing slowly if at all. (d) 200 μM (*S*)‐DHPG blocked 1 mM glutamate‐mediated enhancement (*m* = 7, r = 4), suggesting it may be a neutral antagonist. Box‐and‐whisker plots in (a–d): individual data expressed relative to control grand mean; statistical analysis of raw data: (a–c) one‐way ANOVA with replicates, post‐hoc pairwise paired *t*‐tests, significant differences shown after Benjamini–Hochberg correction method for multiple tests; (d) paired *t*‐tests versus relevant controls (controls not shown).

The non‐selective antagonist (*RS*)‐3,5‐DHPG totally blocks agonism of PLD‐mGluR (Albani‐Torregrossa et al., 1999; Bewick et al., 2005; Pellegrini‐Giampietro et al., 1996; Schoepp et al., 1994). We here tested its action alone and identified the active enantiomer. Racemic (*RS*)‐3,5‐DHPG tended to reduce stretch‐evoked firing but did not reach statistical significance (Figure 3a, muscles (m) = 5, rats (r) = 5). The (*S*)‐enantiomer was ineffective alone even at 200 μM (m = 5, r = 5), but blocked agonism by exogenous glutamate (*P* < 0.001, m = 7, r = 4 Figure 3b,d). Conversely, the (*R*)‐enantiomer alone, regarded as ‘inert’ on classical GluRs, inhibited firing even at 1 μM (*P* < 0.03, m = 7, r = 5; Figure 3c), which is the first report of it having any action.

Table 1 summarizes this pharmacological screening for PLD‐mGluR ligands.

### 3.2 PLD‐mGluR ligand selection and functionalization for PLD‐mGluR screening

**FIGURE 4:**
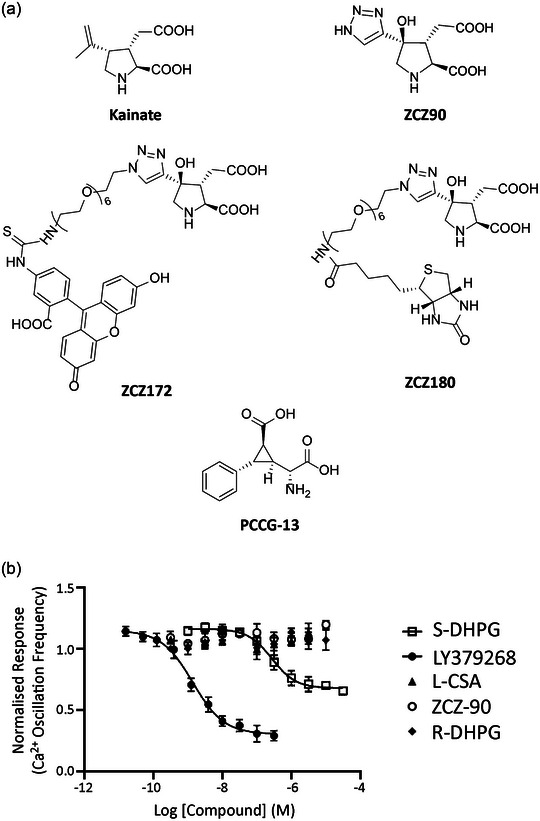
Kainate functionalization and screening ZCZ90 for receptor activity in rat primary cortical neurones. (a) Top: adding a triazole group to kainate produced ZCZ90. Middle: the triazole group enabled a linker side chain to attach either fluorescein (ZCZ172) or biotin (ZCZ180) for use in fluorescence imaging or far‐western blotting, respectively. Bottom: configuration of the structurally highly constrained PLD‐mGluR specific antagonist PCCG‐13, for comparison. (b) FLIPR assay screening of PLD‐mGluR ligands and the functionalized kainate analogue ZCZ90 against glutamate receptors in primary cultures of rat cortical neurones (all *n* = 3). *S*‐DHPG (gp I agonist) and LY379268 (potent gp II agonist) both reduced Ca^2+^ oscillation frequency (EC_50_ 336 ± 213 nM and 1.5 ± 0.27 nM respectively; both *P* < 0.0001; two‐way ANOVA). However, the PLD‐mGluR agonists ZCZ‐90, l‐CSA and the enantiomer (*R*)‐DHPG had no effect (all *P* > 0.5, two‐way ANOVA) indicating they do not activate classical mGluRs. Both kainate and ZCZ90 are PLD‐mGluR agonists, blocked by PCCG‐13 (10 μM; Zanato et al., 2014).

From screening, kainate was chosen for functionalization as other ligands were either too structurally constrained or non‐selective (Figure 4a; Zanato et al., 2014). Functionalization enabled the resulting compound, ZCZ90, to be attached to fluorescein (ZCZ172) or biotin (ZCZ180) by a linker that distanced the functional group from the ligand binding interaction. All three ZCZ compounds retained agonist activity on PLD‐mGluR (i.e., antagonized by PCCG‐13; Zanato et al., 2014).

These compounds were then screened for off‐target effects by fluorescence imaging plate reader (FLIPR) assay using rat cortical neurone primary cultures and AV12 cells expressing cloned human mGluRs 1, 2, 3, 6 or 8. ZCZ90, (*R*)‐DHPG and PCCG‐13 had no effect on either assay, but cells responded strongly to control group (gp) I and gp II agonists (*P* < 0.001, two‐way ANOVA; Figure 4b) and antagonists (Sanger et al., 2013). l‐CSA, a putative endogenous agonist for PLD‐mGluR, produced weak/inconsistent responses in AV12 cells (Supporting information Table S1).

**FIGURE 5:**
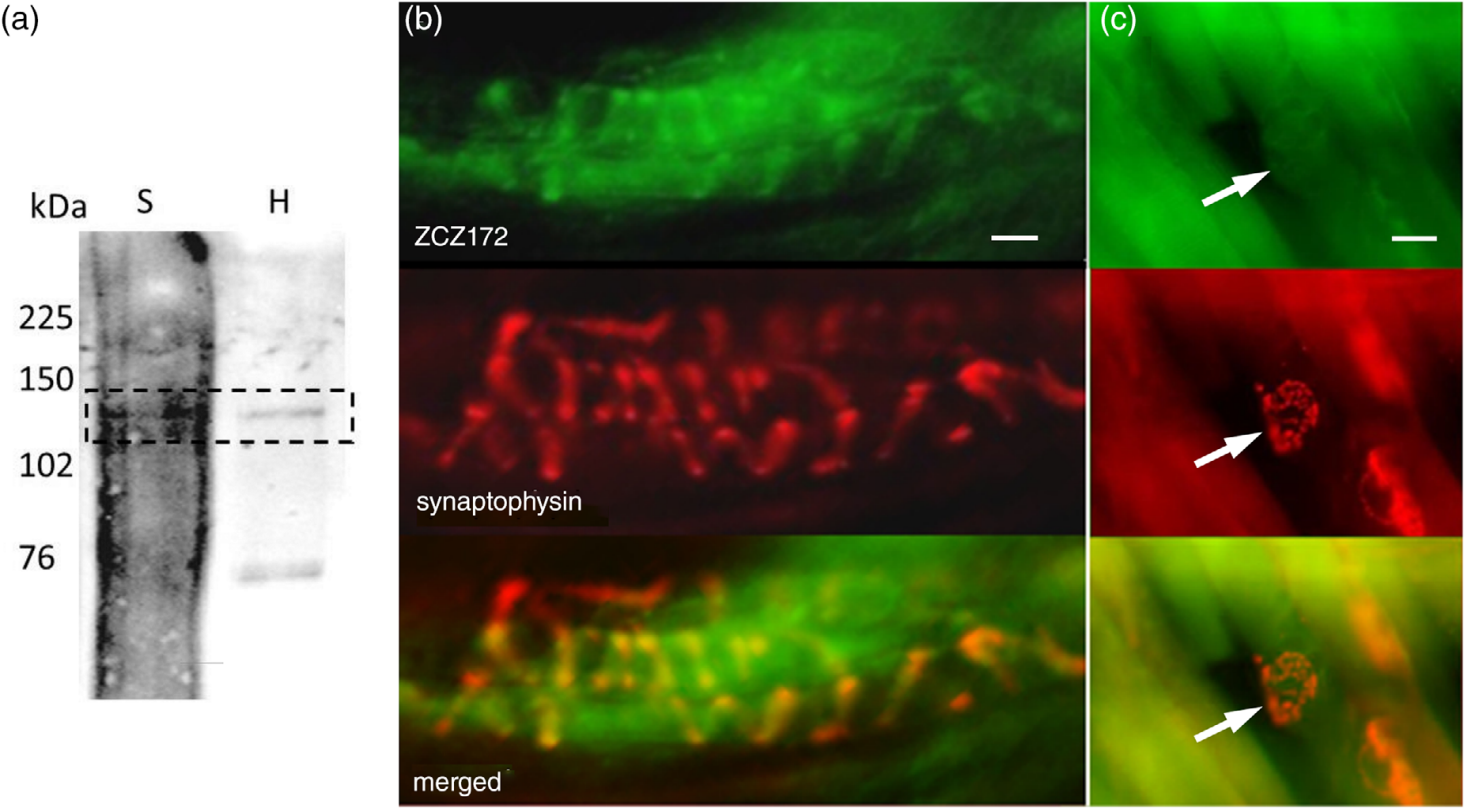
Kainate derivatives ZCZ180 and ZCZ172 label muscle spindle blots and sensory endings, respectively. (a) ZCZ180, the biotinylated kainate derivative, labelled an approximately 120 kDa protein (dashed box) in tissue homogenates of both spindle‐rich material (S), and the hippocampus (H; positive control). (b) In fixed, squashed rat deep lumbrical muscle whole mounts, fluorescently tagged kainate derivative ZCZ172 (green) labelled the sensory nerve endings of muscle spindles, colocalizing (merged) with immunolabelling for synaptophysin (red) in synaptic‐like vesicles in the endings. (c) In contrast, in the same preparations, there was no ZCZ172 signal (green) above background at motor nerve terminals (arrows) on nearby extrafusal fibres. There are three motor terminals in this field identified by anti‐synaptophysin immunofluorescence (red), two (red, upper and lower right) out of the focal plane, on the fibre edges, and one *en face* (centre) in the plane of focus. Note, the high background ZCZ172 labelling reflects the minimal wash, as its binding is readily reversible, unlike antibodies. Images are representative of three separate experiments. Scale bar = 10 μm.

Thus, ZCZ90 seemed only to activate PLD‐mGluR, so ZCZ90‐derived compounds were therefore valid for screening spindle homogenates (western and affinity blot) and to label spindles in whole mount tissues. In far‐western (affinity) blots the biotinylated‐ZCZ90 ligand (ZCZ180) labelled a band of ∼110 kDa, matching a band in the hippocampus positive control (Figure 5a) where PLD‐mGluR pharmacology was first extensively characterised (Pellegrini‐Giampietro et al., 1996). The fluorescein‐linked ZCZ90 analogue (ZCZ172) produced bright fluorescent labelling of spindle sensory terminals (rat lumbrical muscle; Figure 5b). It did not label efferent (motor) terminals at neuromuscular junctions on extrafusal muscle fibres in the surrounding muscle (Figure 5c), identified by their distinctive pretzel‐shaped arborization in synaptophysin immunolabelling of the synaptic vesicles. This indicates selective primary sensory nerve terminal membrane binding by ZCZ172.

### 3.3 PLD‐mGluR is a homomeric kainate GluK2

These data suggest PLD‐mGluR may be a kainate receptor. Indeed, we found mRNA for several GluKs expressed in primary afferent somata (Supporting information Figure S12.1). However, this does not determine whether these are to enable expression in the peripheral sensory terminal rather than the central synaptic ending. Also, the lack of inhibition of glutamate modulation by 1 mM kynurenate (Bewick et al., 2005) or 10 μM 2,3‐dihydroxy‐6‐nitro‐7‐sulfamoyl‐benzo[f]quinoxaline‐2,3‐dione (NBQX) (Zanato et al., 2014) argues against ionotropic kainate receptors, or other ionotropic receptors, being responsible. We, therefore, screened spindle homogenates and whole‐mount muscle labelling using antibodies for all glutamate receptors.

**FIGURE 6:**
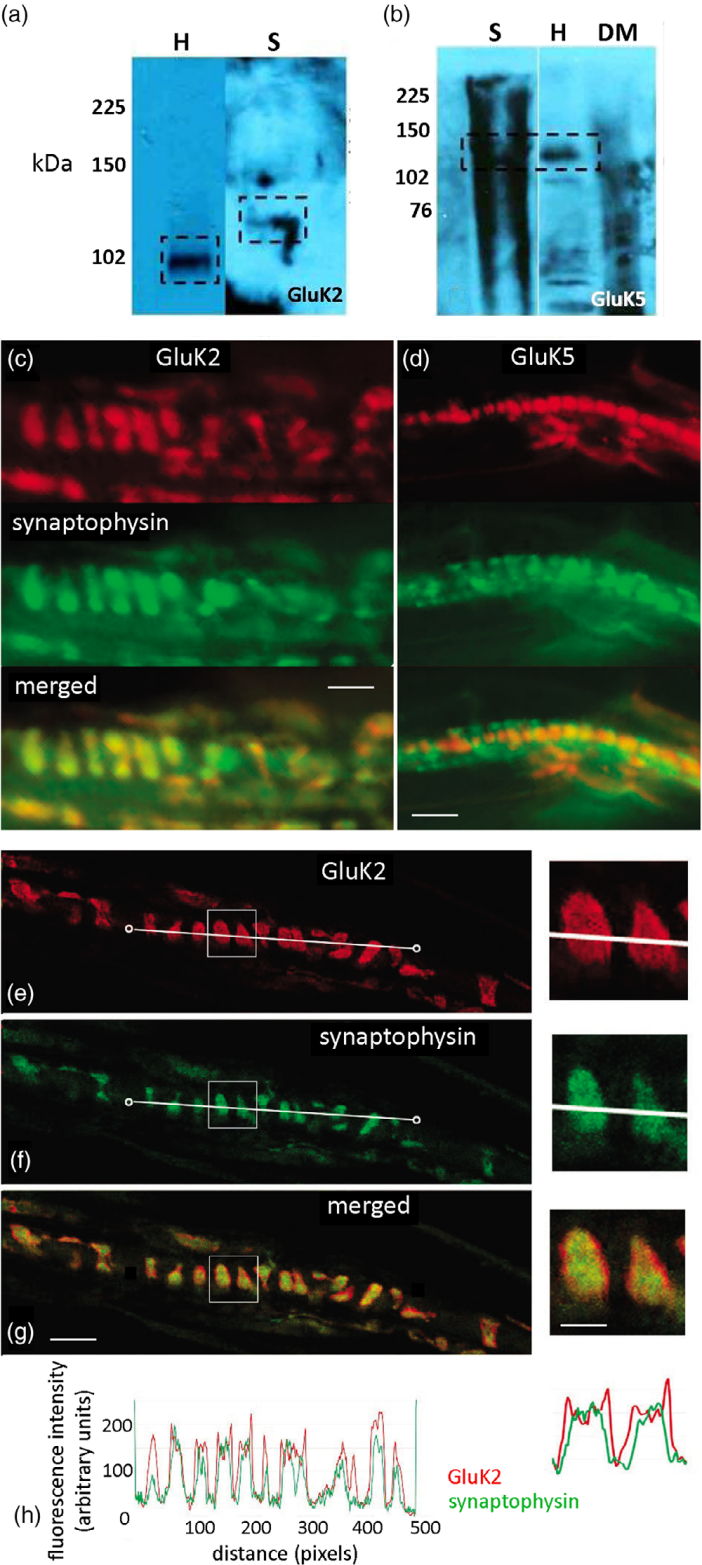
Muscle spindle primary sensory terminals express only GluK2; GluK5 is in intrafusal muscle fibre nuclei. (a, b) Western blots of spindle‐rich homogenate contained bands (boxed areas) for (a) GluK2 (113 kDa; *n* = 5) and (b) GluK5 (123 kDa; *n* = 4). The spindle (S) GluK2 band was slightly heavier than that in the hippocampus (H) positive control. DM, spindle‐free lateral deep masseter (negative control); kDa, kilodalton, marker lane. (c) In teased rat lumbrical whole mount preparations, spindle sensory terminals (green synaptophysin labelling) are brightly labelled by GluK2 antibodies (red). (d) However, GluK5 antibodies (red) labelled only the nuclei of muscle spindle intrafusal muscle fibres. Merged images (lower panels) show these spatial relationships more clearly. (e–g) Confocal optical sections showed a halo of enrichment for GluK2 at the edges of the terminal. Intensity quantification of a line along the central long axis of the terminal helix (white line connecting two circles) for GluK2 (e, red) and synaptophysin (f, green) showed GluK2 labelling present throughout the terminal, but it also extended beyond that of synaptophysin (h), more clearly seen in the enlargements (right). Thus, GluK2 is expressed throughout the terminal, possibly within the SLVs, but particularly highly on the terminal surface. Scale bar = 10 μm main images, 4 μm insets.

Spindle‐free portions of masseter muscle (negative controls) produced no bands for any GluRs (Figure 6a,b). Spindle homogenate generally produced a high background but distinct bands were found at ∼120 kDa for two kainate GluR subunits, GluK2 and GluK5. The GluK5 band matched that in hippocampus but the hippocampus GluK2 band was at a lower molecular mass (∼105 kDa). In whole mounts, GluK2 antibody brightly labelled spindle sensory nerve terminals (Figure 6c,d). In confocal optical sections, GluK2 expression colocalized with synaptophysin, marking SLV clusters within the terminal, but had particularly high expression at the terminal surface (Figure 6e–h). In contrast, GluK5 labelling in confocal images was only in the nuclei of intrafusal muscle fibres. GluK1, 3 and 4 antibodies produced very faint bands in spindle homogenates, and no immunofluorescence above background in muscle whole‐mounts (data not shown).

**FIGURE 7:**
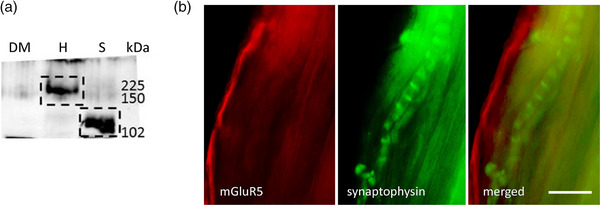
mGluR5 is expressed on non‐spindle sensory axons. (a) A band for mGluR5 was present in spindle homogenates (S) but at a substantially lower molecular mass than in hippocampus (H, positive control). No bands were detected in lateral deep masseter (DM; negative control). (b) In whole‐mounts, the mGluR5 antibody (red) labelled thin, unmyelinated axons (arrow), presumptive nociceptive sensory axons, near spindle capsules but not primary mechanosensory terminals (green, arrow). The merged image shows the separate locations more clearly. Scale bar = 10 μm.

Spindle homogenates also had an mGluR5 band (Figure 7a) at 102 kDa, which was lighter than in hippocampus controls. In muscle whole‐mounts, mGluR5 antibodies labelled only small‐diameter nerve fibres adjacent to, or within, the external spindle capsule (Figure 7b,c). No other bands or labelling was found for any other mGluR, or the NMDA GluN1, found in all NMDA receptors (NMDA‐Rs; Béhé et al., 1995), or AMPA GluA2/3 subunits, found in almost all AMPA receptors (AMPA‐Rs; Greger et al., 2002; Wenthold et al., 1996) (data not shown).

Thus, as the only subunit detected, PLD‐mGluR in muscle spindle primary sensory terminals seemed to be a homomeric GluK2 receptor.

#### 3.3.1 GluK2 glutamate‐mediated signalling in spindles is purely metabotropic

**FIGURE 8:**
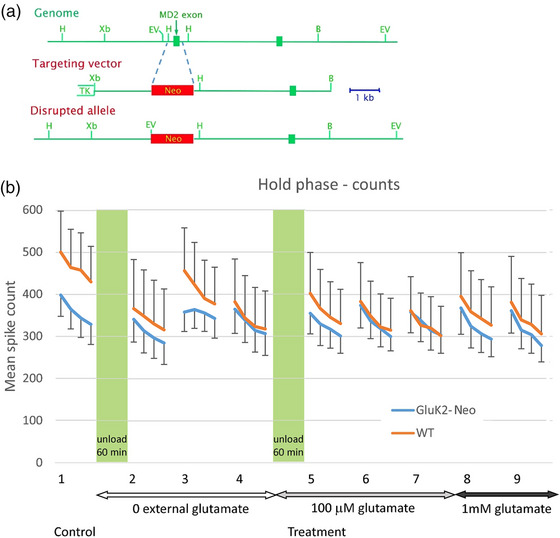
Glutamate‐induced modulation of muscle spindle firing is still present in mice with ablated ionotropic GluK2 function. (a) Schematic diagram showing homologous replacement of the MD2 exon of Grik2 with a Neo‐cassette to disrupt GluK2 ionotropic function (only relevant portion of Grik2 shown). (b) Mean whole‐nerve action potential (spike) counts from mouse soleus muscle at hold‐phase stretch of 1 mm (∼10% initial length) in GluK2‐Neo mice (*n* = 9, m = 5) and wild‐type (*n* = 8, m = 5) littermates. Both genotypes displayed repeatable responses to the first four stretches (group 1 on x‐axis). Also, unloading muscles (60 min), minimizing stretch‐evoked release of endogenous glutamate, markedly inhibited their firing (group 2; both *P* < 0.01). Both recovered strongly (group 3) in the next series, as stretch re‐established endogenous release levels. Adding exogenous glutamate (100 μM) during unloading prevented the unloading‐induced reduction (WT *P* = 0.7; Neo *P* = 0.5). There was no further enhancement to futher increase in glutamate (1 mM) in either genotype. Overall, in both genotypes, glutamate sensitivity in both directions (reduced endogenous release reduced firing, 100 μM exogenous glutamate prevented this) remained. Since GluK2‐Neo ablates ionotropic function, the remaining responses seem most likely to reflect continued metabotropic signalling.

We next tested if ionotropic function was necessary for PLD‐mGluR/GluK2 signalling by testing glutamate pharmacology of soleus muscle spindle firing in a mouse line with GluK2 ionotropic function ablated. In this line, a Neo cassette inserted into the pore‐forming transmembrane MD2 region blocks pore function (Figure 8a), and was termed GluK2‐Neo.

Mean hold phase spike counts for trapezoidal stretches, delivered in groups of four (Figure 8b) and the effects of a variety of manipulations to modulate endogenously‐ and exogenously‐derived glutamate signalling were ascertained. Initial recordings (group 1) showed muscle spindles in WT (*n* = 8, mice, m = 5) and GluK2‐Neo (*n* = 8, m = 5) had broadly similar firing patterns, although both genotypes showed considerable individual variability. A 1 h total unload (muscles relaxed to their intrinsic resting length) was then imposed to minimize stretch‐evoked endogenous glutamate release from sensory terminals. This significantly reduced mean firing in WT (*P* < 0.001) and, to a lesser extent, GluK2‐Neo (*P* < 0.005) muscles (group 2). Resuming stretching plus retaining muscles at in vivo resting lengths between stretch groups, re‐activated endogenous release. This increased (group 3) then decreased (group 4) firing, although it reached statistical significance only for the WT (full statistical analysis given in Supporting information Table S4). Exogenous glutamate (100 μM) prevented the unload‐induced reduction in mean firing (group 5; *P* = 0.5 WT, *P* = 0.7 Neo), consistent with glutamate‐mediated signalling being similar in both genotypes. For the final two groups, increasing the glutamate concentration further (1 mM), produced little further change in mean firing rate for either genotype (all non‐significant), suggesting the system was saturated by 100 μM and/or endogenous release.

Overall, therefore, ablation of GluK2's iGluR function did not prevent GluK2‐Neo mice from responding to endogenous or exogenous glutamate, although responses were somewhat attenuated. This indicates glutamate‐mediated receptor signalling continued and must therefore be metabotropic.

#### 3.3.2 GluK2‐Neo receptor is expressed in spindle sensory terminals, but GluK2 C‐terminus labelling is disrupted

We hypothesized glutamate responses might be attenuated due to the Neo insertion disrupting either GluK2‐Neo expression or the intracellular (C‐terminus) structure. To test which of these were correct, we used ratiometric analysis of immunofluorescence labelling intensities from antibodies to N‐ and C‐terminus epitopes of the GluK2 protein. Labelling intensities in each specimen were normalized to that for synaptophysin in the same soleus muscle spindle terminal, to control for between‐antibody and technical variability.

**FIGURE 9:**
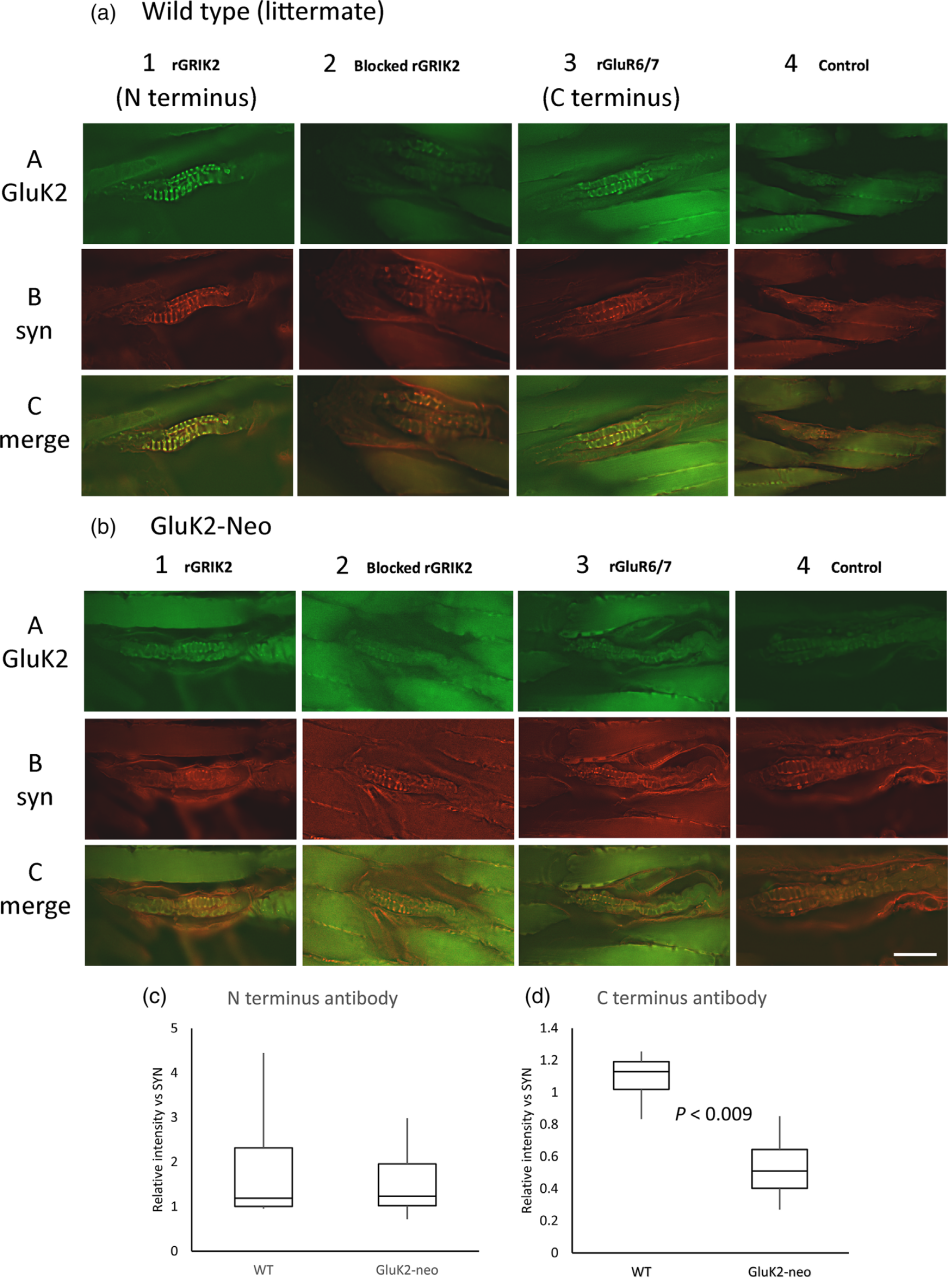
Normal GluK2 expression in soleus muscle spindles of GluK2‐Neo mice but C‐terminus immunolabelling is disrupted. (a, b) Wild‐type (a) and GluK2‐Neo (b) mouse soleus muscle‐spindle primary endings displayed GluK2 immunolabelling with antibodies directed at either N‐terminus (rGRIK2, column 1A, green) or C‐terminus (rGluR6/7, column 3A, green) epitopes, and both colocalized (yellow, 1C, 3C) with anti‐synaptophysin labelling (red, 1B, 3B), a marker for the primary sensory terminals. In both genotypes, labelling was low/absent if primary antibody was pre‐incubated with blocking peptide (rGRIK2, A and B column 2) or the primary antibody was omitted (rGluR6/7, A and B column 4). Scale bar = 100 μm. Thus, full‐length GluK2 is expressed in both genotypes. (c) However, while N‐terminus‐directed antibody (upstream of the insertion) labelling was not different from that in wild‐type mice (*n* = 9), the intensity with C‐terminus‐directed antibodies (downstream of the insertion) was approximately 50% lower in GluK2‐Neo mice (*n* = 7, *P* < 0.009, two‐tailed *t* test for 7 df and α = 0.025 with Bonferroni correction, carried out on arctan transformed data), indicating the intracellular epitope recognized by the antibody was disrupted.

WT spindle sensory endings (*n* = 9 muscles) were labelled robustly with both N‐terminus (rGRIK2) and C‐terminus (rGluR6/7) antibodies (Figure 9a). Similarly, in GluK2‐Neo (*n* = 7 muscles), N‐terminus labelling was unaffected (Figure 9b,c). However, C‐terminus labelling (downstream of the Neo insertion) was reduced by approximately 50% (*P* = 0.009; Figure 9d), indicating lower antibody affinity downstream of the Neo insertion, presumably due to its insertion resulting in structural disruption.

#### 3.3.3 GluK2 is expressed in other mechanosensory terminals exhibiting glutamate‐sensitive function

**FIGURE 10:**
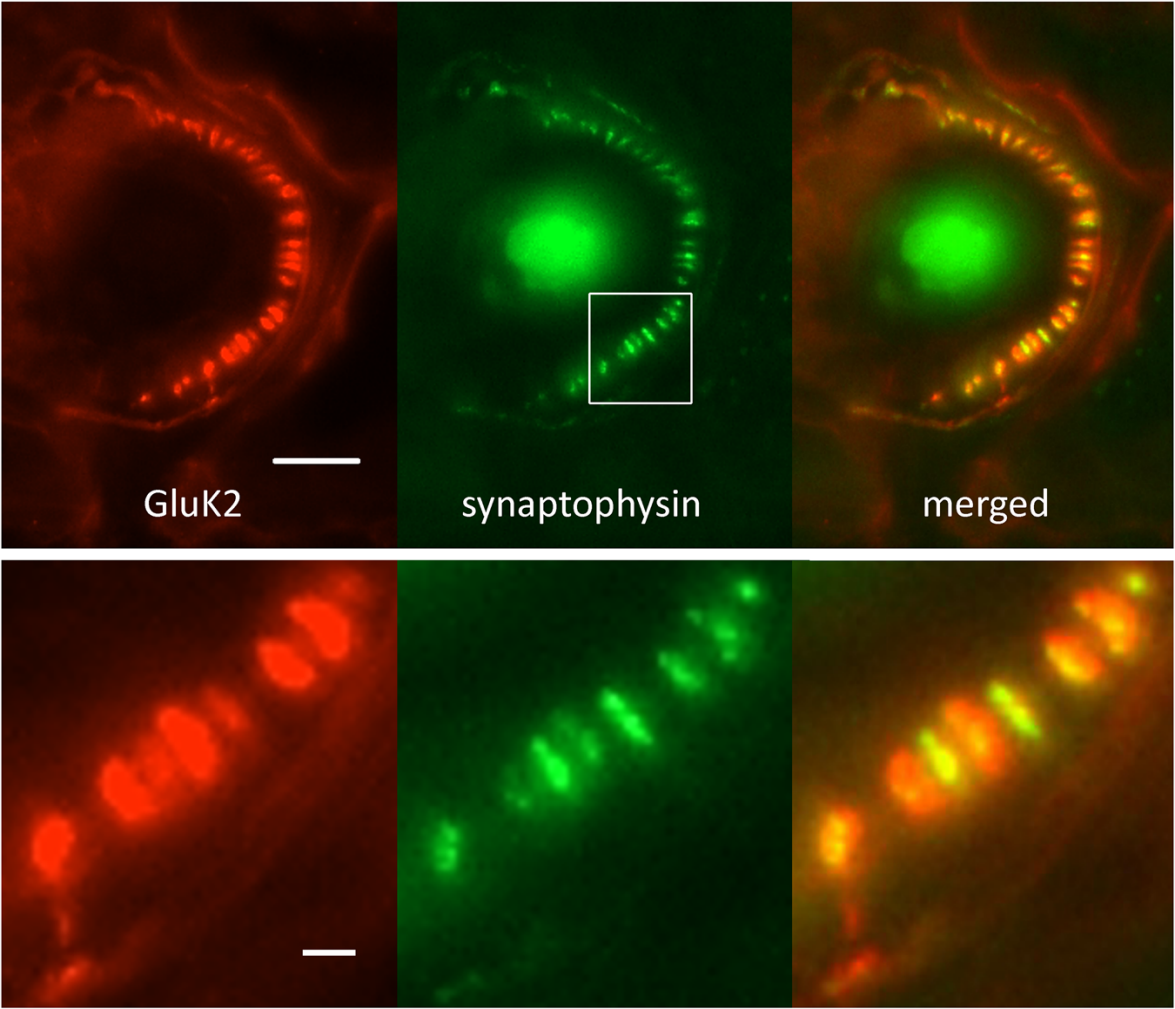
Primary afferent terminals in other mechanosensory organs also exhibit GluK2 immunoreactivity. Adult male Wistar rat ear hair follicle afferents (synaptophysin, green) labelled with antibodies to GluK2 (red). As for spindle primary afferents, the great majority of individual terminals exhibited a halo of red GluK2 labelling surrounding the green core (merged, and enlargement of boxed area), again suggesting a surface membrane accumulation in the terminal. Scale bar = 10 μm (main image) and 2 μm (enlarged).

We have previously shown that PLD‐mGluR also regulates function in hair‐follicle lanceolate endings (Banks et al., 2013; Bewick, 2015), so we tested whether they also express GluK2. Robust labelling was indeed present (Figure 10) and also colocalized with synaptophysin immunofluorescence of SLVs. GluK2 labelling was again brighter at the hair follicle sensory terminal perimeter indicating, as in spindle primary terminals, an elevated expression on the surface membrane.

## 4 DISCUSSION

Overall, these data indicate the PLD‐mGluR is a novel configuration of a known glutamate receptor subunit, that is, it is a homomeric GluK2 receptor signalling purely metabotropically. This conclusion is based on the following observations:

- its atypical pharmacology (i.e., distinct from gp I–III mGluRs, NMDA‐R, AMPA‐R or ionotropic kainate receptor (KAR));
- kainate‐derived functionalized ligands activate spindles but not classical GluRs in vitro;
- kainate‐derived functionalized ligands label annulospiral sensory terminals;
- kainate‐derived functionalized ligands label homogenates from hippocampus, where the PLD‐mGluR was first discovered, and spindles;
- immunoscreening showed GluK2 is the only GluR expressed in the sensory terminal;
- ablating GluK2's ionotropic function does not prevent glutamate‐mediated responses;
- other mechanosensory endings with PLD‐mGluR pharmacology also label for GluK2.

Thus, we conclude PLD‐mGluR is homomeric GluK2, which plays a ubiquitous role in glutamatergic modulation of primary mechanosensory terminals.

### 4.1 Mechanosensory responsiveness and the role of GluK2

How low‐threshold mechanosensory nerve endings transduce stimuli remains poorly understood. Piezo2 is certainly necessary for stretch sensitivity in mammalian muscle spindles (Woo et al., 2015). However, it is unclear if it is sufficient, since its expression levels, pharmacology, ionic conductance and kinetic properties map poorly onto the known properties of the receptor potential, unlike DEG/ENaCs (Bewick & Banks, 2021). Moreover, even in the continued expression of Piezo2, we show here spindle responsiveness is impaired simply by reducing endogenous glutamate release by unloading. Conversely, responsiveness is maintained if glutamate is added during the unloading. Moreover, any interference with GluK2 activation (reducing endogenous glutamate release, blocking SLV exocytosis, blocking the PLD‐mGluR) profoundly inhibits, and can even ablate, firing (Bewick et al., 2005). Clearly, there is a functional necessity for continuous autogenic GluK2 activation of sensory terminals to maintain stretch sensitivity regardless of Piezo2 expression (Bewick, 2015; Bewick & Banks, 2015, 2021). However, the glutamatergic system's involvement cannot be as a step in a linear model of:

Stretch→Piezo2 opening→glutamate release→GluK2 activation→PLD activation→firing.

Spindle firing initiates within milliseconds of stretch stimulation but glutamate‐mediated metabotropic effects take many minutes to appear and often hours to maximize. This is consistent with receptor signalling metabotropically via PLD, which is simply too slow to be involved in directly triggering stretch‐evoked firing.

Rather, the model emerging is that GluK2 mediates an automatic gain‐control mechanism within the sensory terminal regulating the availability or responsiveness of the transducer element(s) (Bewick & Banks, 2015, 2021; Than et al., 2021). Thus, glutamatergic signalling is permissive for stretch‐responsiveness and for regulating the quantity of firing induced but not as the initial trigger for stretch‐evoked firing.

This receptor has been little studied previously, perhaps reflecting the difficulty in isolating its actions in the central nervous system (CNS) amongst all the many other GluRs present (Krishnan et al., 2016). The findings in the present study shows the PLD‐mGluRs in spindles and hippocampus are very similar. These data extend the burgeoning evidence that the pharmacology of the PLD‐mGluR differs substantially from the canonical ionotropic or metabotropic GluRs (Banks et al., 2002, 2013; Bewick, 2015; Bewick et al., 2005). Rather, it very much resembles that of the atypical glutamate receptor directly linked to PLD best characterized in the adult rat hippocampus (Pellegrini‐Giampietro et al., 1996). They also extend the observation that the specific inhibition of the receptor by PCCG‐13, and now (*R*)‐3,5‐DHPG, is a defining receptor characteristic. The present study then identifies the PLD‐mGluR as a homomeric GluK2 receptor, independent of other iGluRs and mGluR subunits.

### 4.2 Only one GluR is expressed in the primary mechanosensory terminal

GluK2, GluK5 and mGluR5 were detected in spindle homogenates, but only GluK2 was in spindle mechanosensory terminals. It is not clear why in western blots GluK2 ran at a lower molecular mass in hippocampus than spindles, although it may be because of splice variants since there are 41 known transcripts of the GRIK2 gene (Ensembl database). Further experiments are needed to determine if there are different splice variants in these locations. Regardless, only GluK2 immunolabelling was found on spindle sensory terminals. The GluK5 labelling in intrafusal muscle fibre nuclei is curious. It may, perhaps, be an artefact of antibody non‐specific cross‐reactivity. Alternatively, it may be related to the expression of Piezo2 in these nuclei (Kim et al., 2020). Again, further experiments are needed to unravel this curious observation. The mGluR5 labelling on unmyelinated axons adjacent to spindles supports its potential role in muscle spindle‐associated nociception (Banks et al., 2013; Lund et al., 2010). The pharmacology of the spindle mechanosensory response modulation is quite distinct from that of mGluR5, and we found no mGluR5 labelling in the spindle sensory endings, unlike in a previous study (Lund et al., 2010). We found the mGluR5 in spindle homogenates had a smaller molecular mass in western blots, suggesting nociceptors express a truncated isoform which, together with antibody specificity, may explain this discrepancy. Overall, therefore, GluK2 is the only GluR expressed in primary mechanosensory endings of muscle spindles, indicating they are a good system for studies of native GluK2/PLD‐mGluR properties in situ.

### 4.3 GluK2 in spindles is metabotropic

It is interesting that the iGluR antagonists kynurenate and NBQX do not block the enhancement by glutamate. This was the main evidence to date that the spindle PLD‐mGluR's action was independent of any ionotropic functionality. The continued glutamatergic responses in the GluK2‐Neo mice with ionotropic action ablated (Mulle et al., 1998) now confirms this conclusion. The slightly blunted responsiveness likely reflects the downstream structural disruption from the insertion of the Neo cassette into the N‐terminus.

It is becoming well established that kainate receptors can signal metabotropically. They still modulate neuronal excitability during ionotropic blockade, downregulating hippocampal GABA signalling (Rodríguez‐Moreno & Lerma, 1998). Moreover, this signalling is not pertussis toxin sensitive, showing it is not G_i/o_ coupled (Rodríguez‐Moreno & Lerma, 1998), consistent with a PLD‐signalling linkage in these cells, too. They also modulate both presynaptic glutamate release and postsynaptic inhibition of slow after‐hyperpolarization (sAHP) in hippocampal CA3 pyramidal cells (Rodrigues & Lerma, 2012). Other PLD‐mGluR properties also support the proposition they are a kainate receptor. KARs recycle both to and from the surface membrane in vesicles, while KAR surface expression, at least in hippocampal neurons, is dependent on metabotropic autoregulatory feedback driven by external concentrations of kainic acid (González‐González & Henley, 2013). This arrangement is again reminiscent of SLV recycling properties in lanceolate endings of hair follicles, where their recycling increases with exogenous glutamate activation of the PLD‐mGluR, an effect blocked by PCCG‐13 and PLD inhibitors (Banks et al., 2013). It is also striking that these metabotropic hippocampal kainate receptors contain GluK2, but not GluK1 (Fisahn et al., 2005). In hippocampal pyramidal cells, heteromeric receptors have separable ionotropic and metabotropic signalling properties (Ruiz et al., 2005). In these GluK2/GluK5 receptors, kynurenate blocks only the fast excitatory postsynaptic currents, not the sAHP. Conversely, GluK2 knock out selectively ablates the sAHP, indicating its importance for metabotropic functionality. Another parallel is that the metabotropic sAHPs in hippocampal neurones are mediated by Ca^2+^‐activated K^+^‐channels, also highly expressed in muscle spindle and lanceolate primary mechanosensory endings (Shenton et al., 2014). Thus, there are strong similarities between the functions we propose for PLD‐mGluR in primary mechanosensory endings and these demonstrably GluK2‐containing kainate receptors in the CNS. An important distinction, however, is that in the spindle we found no evidence for other GluK subunits, or indeed subunits of other GluRs. Therefore, the spindle GluR is a homomeric GluK2. However, it is possible other, less well characterized, GluRs such as δ‐GluRs (GluDs) (Orth et al., 2013), may be present.

Given that GluK2 can form homomeric ionotropic channels, the proposed purely metabotropic action here is still curious. It may be that in the sensory terminal GluK2 does not function as a tetramer. Rather, since formation of many iGluRs is via a ‘dimer of dimers’ association, at least in expression systems (Fisher & Fisher, 2014), it may be that purely metabotropic GluK2 stabilizes at the dimer stage. This could enable the metabotropic functionality without ion channel formation. The present data seem to require some such mechanism.

### 4.4 ZCZ90 and (*R*)‐3,5‐DHPG as novel selective GluK2 ligands

How GluK2 signals metabotropically is unknown. A major factor impacting studies is the lack of GluK2‐specific ligands (Pinheiro & Mulle, 2006). In the present work, the FLIPR study screening on primary cortical neurones, in which (*R*)‐3,5‐DHPG and ZCZ90 provoked no response and thus might be specific for homomeric GluK2, suggests these may be important ligands, in addition to PCCG‐13, for such studies.

### 4.5 How might GluK2 link to PLD

The mechanism linking PLD‐mGluR to PLD activation is unknown. mGluRs commonly activate PLD via protein kinase C as well as G‐proteins of the ADP‐ribosylation family (Servitja et al., 2003). This is also true of hippocampal PLD‐mGluR in neonatal rats (Klein et al., 1997) but the PKC‐dependence is lost in adults (Pellegrini‐Giampietro et al., 1996). Indeed, GluK subunits do not contain a conventional G‐protein coupling motif (Contractor et al., 2011). However, GluK2 interacts strongly with the neuropilin‐ and tolloid‐like proteins (Neto1 and 2) and, thereby, the PDZ scaffold component glutamate‐receptor interacting protein (GRIP) (Tang et al., 2012). When co‐expressed in HEK293 cells, Neto2 slows the onset of desensitization of GluK2, increases its recovery rate and enhances its glutamate sensitivity (Fisher, 2015). Conversely, GluK2 deletion decreases expression of Neto1 in the hippocampus of mice (Straub et al., 2011) and, perhaps particularly relevant to the current study, metabotropic kainate receptor signalling is reduced in Neto1 knock out mice (Wyeth et al., 2014). Thus, GluK2 metabotropic signalling seems likely to involve Neto proteins. This interaction is conserved even when the GluK2 N‐terminal domain is deleted, indicating multiple strong interactions along the length of the receptor (Li et al., 2019) and explaining the persistence of metabotropic functioning in the GluK2‐Neo mouse muscle spindles seen in the present study. Thus, GluK2's metabotropic actions likely require intermediary proteins, perhaps via Neto1 and GRIP. Since GRIP is essentially a structural receptor trafficking protein, how it or Neto might interact with the PLD signalling pathways is unclear.

### 4.6 GluK2 in other primary mechanosensory endings

We have previously proposed that PLD‐mGluR‐mediated modulation is a ubiquitous feature of primary mechanosensory nerve terminals, evidenced ultrastructurally by the presence of SLVs in all such endings (Bewick et al., 2005; Cauna, 1966; Katz, 1966) and the glutamate, kainate and/or PCCG‐13‐sensitivity of terminal function in all such terminals we have studied (Banks et al., 2013; Bewick et al., 2005). The robust GluK2 immunolabelling found in the lanceolate endings supports this proposed ubiquity of this modulatory system, and that GluK2 is the PLD‐mGluR mediating the signalling.

In summary, these data provide strong evidence that GluK2 is the molecular correlate of the PLD‐mGluR activity mediating the glutamatergic modulation of mechanosensory ending firing. As the only detectable GluR present, it seems it is this GluK2 which is blocked by PCCG‐13, a highly selective PLD‐mGluR antagonist. We suggest its purely metabotropic function may indicate GluK2 subunits act in a dimeric configuration. Such a novel arrangement would explain its novel functionality. A homomeric GluK2 receptor would explain its highly atypical pharmacology and its resistance to characterization until now. These findings, therefore, not only significantly progress this receptor's characterization but also indicate primary mechanosensory terminals as powerful systems for studying the function of the native receptor in situ.

## AUTHOR CONTRIBUTIONS

Specific aspects of the work were performed in the following laboratories: G.S.B.: electrophysiology, western blotting, far‐western/affinity blotting, whole‐mount immunolabelling and affinity labelling, and associated imaging. S.P.: C‐ and N‐terminus labelling of GluK2‐Neo muscles. J.dN.: in situ hybridization of DRGs. MZ: kainate functionalization. Eli Lilly: FLIPR assay. All authors contributed to: conception or design of the work; acquisition, analysis or interpretation of data for the work; and drafting of the work or revising it critically for important intellectual content. All authors have read and approved the final version of this manuscript and agree to be accountable for all aspects of the work in ensuring that questions related to the accuracy or integrity of any part of the work are appropriately investigated and resolved. All persons designated as authors qualify for authorship, and all those who qualify for authorship are listed.

## CONFLICT OF INTEREST

H.E.S., B.A.H., L.M.B. and D.B. were employees of Eli Lilly at the time the work was done, and Eli Lilly joint‐funded the scholarship for S.W.’s contribution to this study.

## Supporting information

Supporting information
