# Supplementary material for: The atypical ‘hippocampal’ glutamate receptor coupled to phospholipase D that controls stretch‐sensitivity in primary mechanosensory nerve endings is homomeric purely metabotropic GluK2
Source: Exp Physiol. 2023 Sep 1;109(1):81–99. doi: 10.1113/EP090761 (PMC10988755; doi:10.1113/EP090761)
Supplement: Supplementary file 1 — Supporting information [file EPH-109-81-s001.docx]

**Supplementary Information 1**

**Methods**

**S1 - Animals**

All animal work was in accordance with the UK Animals (Scientific Procedures) Act, 1986, Amendment Regulations, 2012, and approved locally by the University of Aberdeen Animal Welfare and Ethical Review Board. Rats (adult Sprague-Dawley, either sex and >250gm) and mice (GluK2-Neo, with a Neo cassette disrupting expression) were housed at 19-23°C, in 45-65% humidity, on corncob, wood shaving and paper wool bedding, 12hr on/off day/night cycle, with *ad-libitum* water and food (Special Diet Services, UK). Environmental enrichment included cardboard tubes and hanging mouse retreats. Housing was up to 3 (rats) or 10 (mice) animals per box. For acute experiments, animals were killed humanely by species, weight and age-appropriate approved Schedule 1 methods before tissues were removed. Specific details of killing methods are given at the appropriate point in each section of the methods. GluK2-Neo mice were generated as previously described (Mulle *et al*., 1998), in which a PGK-neoR cassette replaces the *Eco RV/Stu I* fragment (687 bp) that overlaps MD2 pore-forming domain of the GluK2 gene on an 129Sv mouse background, which ablates the formation of the ionotropic pore. Unless otherwise stated, rats were killed by exposure to rising CO_2_ levels until cessation of heartbeat, while mice were killed by cervical dislocation. Both methods were followed by decapitation to ensure cessation of life.

**S2 - Electrophysiology**

Electrophysiological recording of stretch-evoked spindle responses from rat lumbrical muscles (Bewick *et al.*, 2005) and mouse soleus muscles (Lin *et al.*, 2016) were as described previously. Briefly, nerve-muscle preparations were dissected and placed in gassed (95% O_2_/5% CO_2_) physiological saline at room temperature (17-20°C), then the evoked electroneurogram was recorded by laying the nerve across silver wire electrodes (rat lumbrical) or taking it into a glass suction electrode (mouse soleus). During dissection, before recording and between recordings the muscles were held at a totally flaccid length, with no tension applied. During recordings, muscle initial length was set such that shortening produced no diminution of afferent firing, but lengthening reliably produced an immediate increase in afferent action potentials. Ramp-hold-release stretches were applied with an increase in length of 1mm. This corresponds to 10% increase in length of both rat lumbrical and mouse soleus muscles, which are approximately 10mm in length *in vivo* (range 9-12mm across all muscles used). Signals were amplified (Rat: A103, Isleworth Electronics, Isleworth, UK and 8102, CF Palmer, High Wycombe UK preamplifiers in series. Mouse: Neurolog NL104 AC pre-amplifier, Digitimer, UK), displayed on an oscilloscope (DSO 400, Gould, Diss, UK) and captured to computer hard drive (WCP software, John Dempster, University of Strathclyde, UK). It was then converted to Spike2 (CED, Cambridge, UK) for further processing and analysis.

**S3 - Deep masseter dissection, cryo-sectioning, spindle column localisation and 3D reconstruction**

In most mammalian skeletal muscles, muscle spindles occur singly, and are widely dispersed (Banks, 2006). However, the rat deep masseter muscle, unlike guinea pig, rabbit, cat and macaque monkey (Rowlerson *et al*, 1988), is an exception to this general pattern. The spindles are reported to be clustered in a single column close to the anterior margin (Karlsen, 1965; Maier, 1979; Rowlerson *et al*, 1988). To verify this and determine the exact location of the spindle column for dissection, the rostral part of the rat deep masseter muscle (Figure S3.1a) was removed and divided into three approximately 5 mm wide longitudinal strips, snap-frozen in 2-methylbutane (Sigma-Aldrich, UK) cooled over liquid nitrogen and stored at -70°C until use. Transverse sections (10 µm thick; 1850 UV cryostat, Leica, Germany) were taken. Sectioning and haematoxylin and eosin staining was carried out at the Microscopy and Histology Facility at the University of Aberdeen. 1 in 40 sections were mounted onto Superfrost polylysine coated slides (Fisherbrand, United Kingdom). As the muscle spindles are 0.7-1.6 mm in length, this was considered sufficient to ensure that all spindles were captured in at least one section in the series. Slides were air dried for 20 min, then stained with haematoxylin and eosin and coverslip applied (Figure S3.1b).

Stained sections were viewed on a modified Nikon Optiphot 2 upright microscope (Micro Instruments Ltd, UK) and images captured using x3.6 and x10 objectives with a Retiga EXi camera and Volocity software (Perkin Elmer, UK). 3-dimensional modelling was carried out using Reconstruct software2 (NIH Software, USA; <https://www.bu.edu/neural/Reconstruct.html>). The images collected using a x3.6 objective to create a photomontage of each section, and the section montages were aligned. Then, all individual muscle spindle profiles in each section (Figure S3.1b) were outlined manually and compiled by the software across all sections and montages to form a 3D model of muscle spindle location within the tissue (Figure S3.1c).

The 3D reconstructions showed that individual spindles were scattered throughout much of the medial part of the muscle. However, the high concentration cluster was located in a tight column spanning the dorsoventral length of the muscle, at the rostromedial margin (Figure S3.1b,c). Conversely, the rostro-lateral muscle was found to be spindle deficient (not shown), so was used as a negative control when homogenised (see S4).

**Figure S3.1**  **Deep masseter spindle column localisation and dissection.** a) Location of the deep (***Deep***) masseter muscle in the rat, and its column of approximately 40 spindles (red dashed line) close to its rostromedial surface. b) Multiple closely packed spindle clusters (boxes) in a typical H&E-labelled muscle section. Five equatorial spindle profiles are shown, 3 with very prominent (equatorial) and 2 less prominent (near equatorial) capsular spaces. The extensive nearby densely packed smaller intrafusal muscle fibres indicate the many parallel adjacent spindles sectioned at more polar regions whose equators lie above or below the plane of section. c) Reconstruction of H&E-stained skip-serial cryosections of a complete rostral deep masseter muscle. Muscle fibre long axis runs dorsoventrally. Individual spindle equatorial regions are indicated by vertical red lines. The high-density column of ~40 spindles spans the muscle (arrows), parallel to the muscle fibres and lies close to the medial margin, greatly facilitating their dissection, unlike the scattered spindles along the ventral margin. d) Muscle spindle column (arrows) just prior to final isolation by microdissection, identified by its characteristic annulospiral sensory terminal morphology stained with methylene blue. *Image in a) adapted from Sano et al., 2007. J Anat. 211:464–470. doi: 10.1111/j.1469-7580.2007.00783.x. with permission.*

**S4 - Enriched muscle spindle homogenate preparation**

Investigating the PLD-linked glutamate receptor (PLD-mGluR) biochemically required an enriched source of spindle terminal protein. Gentle collagenase digestion (0.1% Type I collagenase, Sigma-Aldrich, UK; 15 min at 37°C in 95% O_2_/5% CO_2_ continuously gassed physiological saline) of muscle strips partially dissociated this column from the surrounding muscle, then methylene blue staining (0.1% methylene blue, 45 min, 37°C in continuously gassed physiological saline) visualised the muscle spindles for isolation by microdissection (Figure S3.1d). Isolated spindle columns and spindle-free rostro-lateral muscle portions, were then homogenised and probed by molecular and biochemical methods.

**S5 - *In situ* hybridisation**

All *in situ* hybridisations were carried out at Columbia University, NY, USA, and in accordance with the Institutional Animal Care and Use Committee of Columbia University regulations. Post-natal age 28 days (P28) mice were anaesthetised by isofluorane until unresponsive to hind foot pinching, then perfusion fixed by cardiac puncture (4mL, 4% paraformaldehyde (PFA)). The spinal cord was removed and fixed (4% PFA overnight at 4°C), then placed in 30% sucrose. Spinal cords were then frozen in Tissue Tek OCT compound (VWR, PA, USA) and stored at -70°C until use.

Primers were designed using NCBI Primer-BLAST, and acquired from Eurofins MWG Operon (KY, USA). Primers were diluted to primer stocks of 100 µM. Primer mixes were subsequently made for each gene to give forward and reverse primer concentrations of 10 µM each.

DNA templates for each gene of interest were made by polymerase chain reaction (PCR) 2x reaction mix (Promega, USA), 0.5 µL M13 forward primer (50 ng/µL), 0.5 µL M13 reverse primer (50 ng/µL), 1 µL (400 nM) target gene primer mix (10 µM), 2 µL cDNA library (embryonic, brain, or DRG), 11 µL ultrapure H2O. DNA was then amplified using the following program, with steps 2-4 repeated for 35 cycles:

1. 94 °C for 5 min

2. Denature: 94 °C for 1 min

3. Anneal: 55-58 °C for 1 min

4. Extend: 72 °C for 1 min

5. Extend: 72 °C for 10 min

6. Hold: 4°C, indefinite

PCR products were then loaded alongside 1.5 kBP ladder onto a 1% Agarose-Tris-Acetate-EDTA (TAE) gel with 7.5 µg ethidium bromide in TAE buffer and separated at 100 V for 45 min. DNA bands were detected on a UV light box (254 nm), bands excised and samples gel purified using a QIAquick Gel Extraction Kit (Qiagen, MD, USA) according to the manufacturer’s protocol. Accuracy in probe production was validated by sequencing digested samples.

Probes were produced by transcription reactions containing: 14 µL gel purified template DNA, 2 µL transcription reaction buffer, 2 µL DIG-labelled nucleotides, 0.5 µL RNAsin, and 1.5 µL T3 or T7 polymerase. Contents were mixed, briefly spun down (approximately 8000 rpm, 15 sec) then incubated at 37 °C for 2.5-3 hr. 3 µL probe was loaded onto a 1% Agarose-TAE gel with 2 µL loading dye and separated at 80 V for 20 min. Bands were excised, gel purified and sequenced as described above, to ensure the appropriate genes would be targeted by probes. The remaining probes were purified using G50 (25)-spin columns according to manufacturer’s instructions (Qiagen, MD, USA). 150 µL hybridisation buffer (50% formamide; 5x saline sodium citrate buffer (SSC; 0.75M NaCl, 75 mM sodium citrate pH 7); 5x Denhardts; 250 µg/µL baker’s yeast RNA; 500 µg/ µL ssDNA) was immediately added.

12 µm serial cord transverse cryosections (1850 UV, Leica, Germany) were mounted (Fisherbrand Superfrost Plus polylysine coated slides, Fisher Scientific, USA), and air dried (20 min) then fixed in 4% PFA in PBS, washed (3x 3 min) in PBS. They were then permeabilised with Proteinase K buffer (6.25 µM EDTA, pH 8.0; 5 µM Tris, pH7.5; 400 µg Proteinase K, 375 mL dH_2_O) for 5 min and the fixation and wash steps repeated. Sections were acetylated (393.3 mL H_2_O; 13.1 µM triethanolamine; 1.3 µM HCl; 1.9 µM acetic anhydride) for 10 min, washed (PBS, 3x 5 min). Sections were prehybridised with 500 µL hybridisation buffer in a horizontal tray, humidified with 50% formamide and 5x SSC (1 hr, room temperature). 2 µL probe was diluted in 100 µL hybridisation buffer, heated at 80°C for 5 min and cooled on ice for 3 min. Hybridisation buffer was removed from slides and replaced with 100 µL of diluted probe. Slides were then cover-slipped and placed in a cassette box humidified with 50% formamide and 5x SSC (separate box for each probe) overnight at 72°C.

Slides were placed in 5x SSC at 72°C and coverslips removed, incubated (72°C, 5 min) in 0.2x SSC (1 hr, 72°C) then 0.2x SSC (5 min, RT), then B1 buffer (0.1 M Tris, pH 7.5; 0.15 M NaCl; 870 mL H_2_O; 5 min RT). Sections were then blocked (B1 buffer with 10% normal goat serum (NGS)) in a humidified (B1 buffer) for 1hr at RT. Blocking solution was removed and sections incubated in anti-DIG antibody (1:5000, in B1 buffer with 1% NGS) in a humidified tray (overnight at 4 °C).

Anti-DIG antibody was washed off (B1 buffer, 3x 3 min), followed by equilibration with B3 buffer (0.1 M Tris pH 9.5; 0.1 M NaCl; 50 mM MgCl2; 332 mL H_2_O; RT, 5 min). Developing solution (15 mL B3, 3 drops levamisol, 15 µL Tween 20 [10%], 52.5 µL BCIP, 67.5 µL NBT) was added to each slide. Slides were then cover-slipped in Dako mounting medium (Agilent Technologies, CA, USA) and developed in a dark chamber (RT, up to 48 hr).

*Imaging:* Sections were viewed with a Nikon Optiphot 2 microscope (Micro Instruments Ltd, UK) and images were captured at x3.6 and x10 objectives with a Retiga EXi camera (Q Imaging, UK) and either AxioVision (Zeiss, Germany) or Volocity software (Perkin Elmer, UK).

**S6 - Spindle immunolabelling and affinity labelling with ZCZ172**

Rat lumbrical muscles were pinned by a piece of the large tendon on their origin (extension of the flexor digitorum longus tendon) and the muscle distal tendon to a PDMS-lined (Sylgard 184 Dow-Corning, Germany) petri dish and fixed in 4% formaldehyde in PBS (Millipore, USA) at 4°C overnight. Tissue was rinsed (3x PBS), cleaned of adherent tissue then teased in PBS with fine forceps on a microscope slide, exposing as many muscle fibres as possible. Muscles were then squashed very firmly with a second microscope slide to burst the capsule surrounding individual muscle spindles and re-pinned in the PDMS-lined petri dish in PBS. Preparations were blocked (1% BSA, 0.4% Triton X-100 in PBS, 1 hr, RT) and incubated in primary antibodies (Supplementary Information 1 Table S2?) in PBS with 1% BSA and 0.1% Triton X-100 (48hr, 4°C). Preparations were then washed (3x 5min, PBS) then incubated in relevant secondary antibody (Supplementary Information 1 Table S3?) in PBS with 1% BSA and 0.1% Triton X-100 in the dark (1hr, RT). Where relevant, the fluorescein-labelled affinity probe ZCZ-172 replaced synaptophysin labelling, by omitting the synaptophysin primary antibody, then adding ZCZ-172 (75µM) to the secondary antibody diluent. Labelled preparations were again squashed between two microscope slides to flatten preparations as much as possible, to ease spindle identification, then floated onto a third microscope slides before mounting (8% Mowiol 4-88, Sigma-Aldrich, UK; 166mM glycerol, 83mM Tris pH 8.5, 223mM 1,4-diazabicyclo(2.2.2)octane (DABCO) and cover-slipped.

*Imaging:* Wide-field images were captured as above, using x10 or x25 objectives, with (red/green emission respectively) excitation wavelengths of 450–490/510–560nm, dichromatic mirror wavelengths of 505/575 nm, and emission wavelengths of 520/590 nm. Confocal microscopy was carried out at the University of Aberdeen Microscopy and Histology Facility on an LSM710 inverted microscope (Zeiss, Germany) with laser excitation wavelengths of 488 (green) and 550 nm (red) and captured using Zen Blue 2012 software (Zeiss, Germany).

**S7 - Immunofluorescence of rat hair follicles**

Adult male Wistar rats were terminally anaesthetised with pentabarbitone and perfused with heparinised saline followed by 4% PFA. Inner ear skin was removed, post-fixed overnight and then transferred to 30% sucrose in 0.1M phosphate buffer (PB) pH 7.4 for a minimum of 24hrs before freezing at -20°C in preparation for collecting 20μm cryosections onto poly-L-lysine coated slides. Nonspecific binding sites were blocked with 10% NGS (Abcam, UK) and 0.1% Triton-X-100 (Tx) in PB for 40 minutes, rinsed in PB (1x 10 min) then incubated in rabbit monoclonal anti-GRIK2 (1:500; Abcam, UK) plus mouse monoclonal anti-synaptophysin (1:200; Abcam, Cambridge, UK) in 1% NGS-0.1% Tx in PB) overnight at 4°C. After washing (x3 in PB) the secondary antibodies, Alexafluor 594 goat anti-rabbit (1:200; ThermoFisher, UK) together with Alexafluor 488 anti-mouse (1:200; ThermoFisher, UK) were applied for 2hrs at room temperature. Following a further washing (x3 in PB), the sections were mounted under Prolong Gold (ThermoFisher, UK) for viewing and image acquisition.

*Imaging:* Sections were examined using an Axioskop 2 (Zeiss, Germany) microscope under epifluorescence. Digital images were captured with an Orca 285 CCD camera (Hamamatsu Photonics, UK) controlled by Improvision Volocity (Acquisition, Restoration and Visualisation) software (v. 6.2.1, Perkin Elmer, UK).

**S9 - Immunofluorescence GluK2-Neo mouse soleus spindles**

Expression of GluK2 was investigated in GluK2-Neo mice. The soleus and splenius muscles were removed from perfused-fixed GluK2-Neo and wild type (WT) mice (n=3 each) and stored (30% sucrose, 4°C). The muscles were embedded in OCT and 20µm longitudinal sections cut using a cryostat, placed on charged slides and stored at -80°C.

Sections were rinsed with PBS, then non-specific binding sites blocked (blocking solution: 10% normal goat serum [NGS; Abcam Ab7481], 4% Triton-X-100 [TX] in PBS) for 30 min at room temperature. The sections were then incubated (1% NGS-01.5TX in PBS) in monoclonal anti-GluR antibodies (Vandenbeuch *et al.*, 2010) specifically rabbit anti-GRIK2 (1:500; Abcam: ab124702) or rabbit anti-GluR6/7 (1:100; Millipore: 04-921) for 48 hrs at 4°C. The GRIK2 antibody binds at the N-terminal end of GluK2 (aka GluR6) while GluR6/7 binds at the C-terminal end, close to the Neo-insertion site. Mouse anti-synaptophysin (SYN; 1:200 Abcam; ab8049) was used to identify muscle spindles within the tissue by labelling the glutamate-containing SLVs in the terminals. After washing in PBS (15 min) the secondary antibodies, Alexafluor 594 goat anti mouse (1:200; Thermo Fisher UK; A-11005) and Alexafluor 488 goat anti-rabbit (1:200; Thermo Fisher UK; A-11008) were applied for 2 hrs at room temperature in the dark. Finally, the sections were rinsed as before and mounted under Citifluor AF4 (Agar Scientific, UK) and stored in the dark at 4°C.

*Imaging:* Labelling was examined using an Axioskop 2 (Zeiss, Germany) under epifluorescence with digital images captured with an Orca 285CCD camera (Hamamatsu Photonics, UK) controlled by Improvision Volocity (Acquisition, Restoration and Visualisation) software (v.6.2.1; Perkin Elmer, UK). Captured images were imported into Photoshop Creative Cloud (v21.2.4; Adobe, CA, USA) to create annotated figures.

**S10 - Preparation of ZCZ172**

***General Information***

All reactions were carried out in oven- or flame-dried glassware under nitrogen atmosphere, unless stated otherwise. All commercially available reagents were used as received. Reactions were magnetically stirred and monitored by TLC on silica gel (60 F254 pre-coated glass plates, 0.25 mm thickness). Visualization was accomplished by irradiation with a UV lamp and/or staining with a ceric ammonium molibdate or KMnO4 solution. Yields refer to chromatographically and spectroscopically pure compounds, unless stated otherwise. 1H (400.13 MHz) and 13C (100.58 MHz) NMR spectra were recorded on a Bruker ADVANCE III spectrometer. 13C NMR spectra were recorded with complete proton decoupling. The solvent resonance was employed as the internal standard H2O  = 4.79 ppm), all the chemical shifts (δ) are expressed in parts per million and the coupling constant (J) are given in Hertz. Multiplicity is described using the following abbreviations: s, singlet; d, doublet; t, triplet; q, quartet; m, multiplet; br, broad signal; or combinations thereof. LC-MS experiments were performed on an Agilent Technologies 1200 Series HPLC system equipped with a DAD and a 6120 MS detector composed by an ESI ionization source and a Single Quadrupole mass selective detector using an Analytical C18 RP Column (Phenomenex Luna, C18, 250x4.60 mm, 5 , 100 Ǻ). HPLC purifications were performed on the Agilent 1200 system using a semi preparative C18 RP Column (Phenomenex Luna, 250x10.00 mm, 5 μ, 100 Ǻ).

***Synthesis of ZCZ172***

***Scheme S10.1.*** *Reagents and conditions: i) CuI, Na ascorbate, DIPEA, DMF, rt, 30 min; ii) H_3_PO_4_ 85%, DCM/CH_3_CN, rt, 15 min (50% in 2 steps); iii) LiOH 2N in water, rt, 6 h; iv) DIPEA, DMF, fluorescein isothiocyanate, rt, 8 h (30% in 2 steps).*

***Synthetic Procedures***

*(2S,3R,4S)-methyl 4-(1-(20-amino-3,6,9,12,15,18-hexaoxaicosyl)-1H-1,2,3-triazol-4-yl)-3-(2-tert-butoxy-2-oxoethyl)-4-hydroxy-1-(9-phenyl-9H-fluoren-9-yl)pyrrolidine-2-carboxylate (****3****)*

To a solution of alkyne **1** (50 mg, 0.09 mmol), CuI (91 mg, 0.48 mmol), sodium ascorbate (713 mg, 3.6 mmol) and DIPEA (1 mL, 7.9 mmol) in DMF (3 mL) a solution of *O*-(2-Aminoethyl)-*O*′-(2-azidoethyl)pentaethylene glycol (**2**, 35 mg, 0.09 mmol) in DMF (1.5 mL) was added. The reaction was stirred at rt for 30 minutes. The resulting mixture was extracted with AcOEt (3 x 10 mL), the combined organic layers were washed with H_2_O (3 x 20 mL), dried over Na_2_SO_4_, filtered and the solvent was evaporated under reduced pressure. The resulting yellow oil **3** (82 mg) was used crude without further purification.

ESI MS *m/z*: [M+H]^+^ calcd for C_48_H_63_N_5_O_11_ 874.4, [M+Na]^+^ calcd for C_48_H_63_N_5_O_11_Na 896.4, found (relative intensity) 874.4 (100) [M+H]^+^, 896.4 (25) [M+Na]^+^.

*2-((2S,3R,4S)-4-(1-(20-amino-3,6,9,12,15,18-hexaoxaicosyl)-1H-1,2,3-triazol-4-yl)-4-hydroxy-2-(methoxycarbonyl)pyrrolidin-3-yl)acetic acid (****4****)*

To a solution of crude compound **3** (82 mg, 0.09 mmol) in a 1:1 mixture of DCM/CH_3_CN (3 mL), H_3_PO_4_ (solution 85% in water, 0.3 mL) was added dropwise. The reaction was stirred for 15 minutes, water (5 mL) was added and the mixture was washed with AcOEt (2 x 20 mL). The aqueous layers were lyophilized and the crude product was purified *via* HPLC (Semi-preparative C18 Luna column, eluent A: H_2_O with 0.1% TFA, eluent B: CH_3_CN with 0.1% TFA, from 0% of eluent B to 30% of eluent B in 30 minutes, retention time: 15.3 min) in order to obtain compound **4** (25 mg, 50% in two steps) as a white solid. ^1^H NMR (400 MHz, D_2_O) *δ*: 2.39 (d, 2 H, *J* = 6.8 Hz), 3.14 (m, 2 H), 3.30 (m, 1 H), 3.61 (m, 20 H), 3.71 (m, 3 H), 3.83 (bs, 3 H), 3.90 (m, 2 H), 4.04 (d, 1 H, *J* = 12.6 Hz), 4.59 (m, 3 H), 8.06 (s, 1 H). ^13^C NMR (100 MHz, D_2_O) δ: 34.3, 39.1, 49.2, 50.1, 53.8, 54.1, 63.4, 66.3, 68.7, 69.5, 69.6, 77.5, 124.9, 145.4, 169.5, 174.2. ESI MS *m/z*: [M+H]^+^ calcd for C_24_H_44_N_5_O_11_ 578, [M+2H]^2+^ 289, found (relative intensity) 578.3 (75) [M+H]^+^, 289.6 (100) [M+2H]^2+^.*(2S,3R,4S)-4-(1-(20-amino-3,6,9,12,15,18-hexaoxaicosyl)-1H-1,2,3-triazol-4-yl)-3-(carboxymethyl)-4-hydroxypyrrolidine-2-carboxylic acid (****5****)*

Compound **4** (25 mg, 0.03 mmol) was dissolved in a solution of LiOH in water (2N, 2 mL). The mixture was stirred for 6 h at rt (progress was checked by mass analysis) then the pH is neutralised by addition of aqueous HCl (1N in water) and the product was lyophilized. The resulting with solid **5** (24 mg) was used crude without further purification.

^1^H NMR (400 MHz, D_2_O) *δ*: 1.78 (dd, 1 H, *J* = 16.2, 9.7 Hz), 2.10 (dd, 1 H, *J* = 16.2, 5.1 Hz), 2.59 (m, 3 H), 3.20 (m, 3 H), 3.35 (m, 2 H), 3.52 (m, 20 H), 3.81 (m, 2 H), 4.45 (m, 2 H), 7.78 (s, 1 H); MS (ESI), calculated *m/z* C_23_H_42_N_5_O_11_ 563.28 [M+H]^+^, found *m/z* (relative intensity) 563.3 (100) [M+H]^+^.

*(2S,3R,4S)-4-(1-(1-(3-carboxy-4-(6-hydroxy-3-oxo-3H-xanthen-9-yl)phenylamino)-1-thioxo-5,8,11,14,17,20-hexaoxa-2-azadocosan-22-yl)-1H-1,2,3-triazol-4-yl)-3-(carboxymethyl)-4-hydroxypyrrolidine-2-carboxylic acid (****ZCZ172****)*

To a solution of crude compound **5** (20 mg, 0.025 mmol) in DMF (2 mL), DIPEA (0.01 mL, 0.05 mmol) and fluorescein isothiocyanate (11 mg, 0.0275 mmol) were added. The mixture was stirred for 8 h at rt (progress was checked by mass analysis) then the solvent was removed and the crude product was purified by HPLC (Semi-preparative C18 Luna column, eluent A: H_2_O with 0.1% TFA, eluent B: CH_3_CN with 0.1% TFA, from 10% of eluent B to 100% of eluent B in 15 minutes, retention time: 13.8 min) in order to obtain **ZCZ172** (7 mg, 30% in two steps) as a white solid.ESI MS *m/z*: [M+H]^+^ calcd for C_44_H_53_N_6_O_16_S 953.32, [M+2H]^2+^ 477.2, found (relative intensity) 953.2 (25) [M+H]^+^, 477.2 (100) [M+2H]^2+^.

**S11 - Resolution of (RS) 3,5-DHPG (2-Amino-2-(3,5-dihydroxyphenyl) acetic acid)**

**Methods**

See main text.

**Figure S11.1.** Structure of (RS) 3,5-DHPG


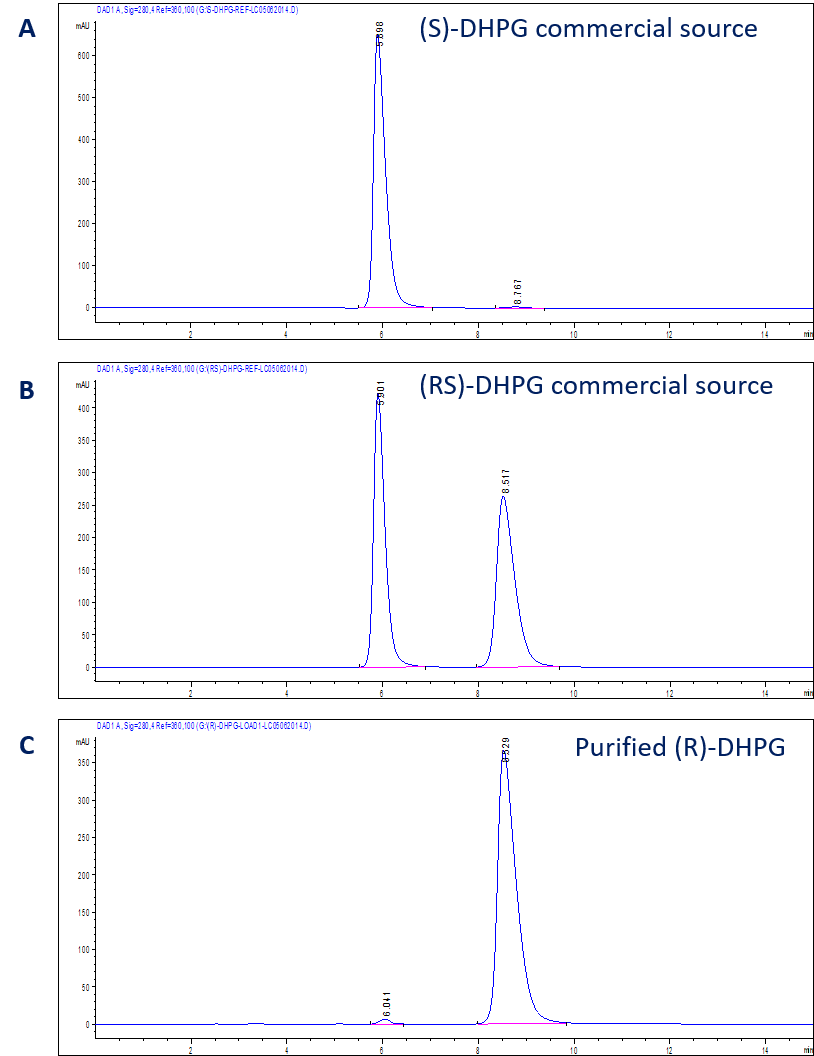


**Figure S11.2.** The peak related to (R)-DHPG was identified by comparison of peaks derived from injection of the racemic mixture and injection of an (S)-DHPG standard. A) Run time (Rt) of (S)-DHPG (Tocris, UK) = 5.9 min. B) Racemic mix displayed the same Rt for (S)-DHPG (left peak) plus a Rt of (R)-DHPG = 8.5 min (right peak. C). (R)-DHPG was obtained with a purity higher than 98%.

Phenomenex Strata-X-C protocol for SI

1. Condition: 2 mL Methanol
2. Equilibrate: 2 mL 0.1 N HCl in water
3. Load: 5 ml diluted sample (N.B. load slowly)
4. Wash: 2 mL 0.1 N HCl in water + 1 mL water
5. Elute: 2 x 1 mL 5% NH_4_OH in Methanol (elute slowly)

**S12 GRIK *in situ*s in mouse DRGs**

**GRIK2**

**Runx3**

**parvalbumin**

**b**

**a**

**c**

**GRIK5**

**GRIK4**

**GRIK3**

**f**

**e**

**d**

**Figure S12.1.** *In situ* hybridisation showed GRIK subunit transcripts in sections of P28 mouse DRGs. DRGs were cryosectioned at 12 μm and probed for transcripts for: (a) parvalbumin and (b) Runx3 as positive controls; then (c) GRIK2; (d) GRIK3; (e) GRIK4; and (f) GRIK5.

**Table S1 EC50/Efficacy data for PLD-mGluR ligands on human mGluRs 1,2,3,6 & 8 in recombinant expression systems.**

* Ca^2+^ mobilisation by FLIPR in recombinant AV12 cells transfected with hmGluR1 and the glutamate transporter.

‡ Ca^2+^ mobilisation by FLIPR in recombinant AV12 cells transfected with either hmGluR2, hmGluR3 or hmGluR8, Ga15 and the glutamate transporter.

† inhibition of forskolin stimulated cAMP in stable human recombinant AV12 cells transfected with hmGluR6 and the glutamate transporter by HTRF.

SP = single point screen.

Values are an n=1 (1 experiment, 3 replicates) relative to a fixed maximal concentration of control agonist (glutamate).

**Table S2 Primary antibodies used in immunoblotting**

| **Antibody** | **Species** | **In TBST** | **Supplier’s Predicted Band Size (kDa)** | **Positive Control** |
| --- | --- | --- | --- | --- |
| mGluR1 (07-617; Merck Millipore [Upstate], Germany) | Rabbit polyclonal | 1:2000 | 142 | Hippocampus |
| mGluR2/3 (06-676; Merck Millipore [Upstate], Germany) | Rabbit polyclonal | 1:3000 | 96 | Hippocampus |
| mGluR4 (ab53088; Abcam, UK) | Rabbit polyclonal | 1:1000 | 102 | Hippocampus |
| mGluR5 (AB5675; Millipore [Chemicon], Germany) | Rabbit polyclonal | 1: 7500 | 132 | Hippocampus |
| mGluR5+1 (ab16214; Abcam, UK) | Rabbit polyclonal | 1:2000 | 125 | Hippocampus |
| mGluR6 (ab10314; Abcam, UK) | Rabbit polyclonal | 1:2000 | 105 | Hippocampus/ Retina |
| mGluR7 (07-239; Millipore [Upstate], UK) | Rabbit polyclonal | 1:500 | 102 | Hippocampus |
| mGluR8 (SAB4501327; Sigma-Aldrich, UK) | Rabbit polyclonal | 1:500 | 101 | Hippocampus |
| GluK1 (ab67317, Abcam, UK) | Rabbit polyclonal | 1:2000 | 104 | Hippocampus |
| GluK2 (ab124702, Abcam [RabMab], UK) | Rabbit monoclonal | 1:5000 | 103 | Hippocampus |
| GluK3 (ab183035, Abcam [RabMab], UK) | Rabbit monoclonal | 1:1000 | 104 | Hippocampus |
| GluK4 (ab67402; Abcam, UK) | Rabbit polyclonal | 1:1000 | 97 | Hippocampus |
| GluK4 (ab67404, Abcam, UK) | Rabbit polyclonal | 1:1000 | 97 | Hippocampus |
| GluK5 (ab67408; Abcam, UK) | Rabbit polyclonal | 1:500 | 95 | Hippocampus |
| GluK5 (ab32672; Abcam, UK) | Rabbit polyclonal | 1:500 | 123 | Hippocampus |
| NR1 (R1JHL/BML-SA493-0015; Enzo Life Sciences, UK) | Mouse monoclonal | 1:1000 | 105 | Hippocampus |
| GluR2/GluR3 (ADI-905-414-1; Enzo Life Sciences, UK) | Rabbit polyclonal | 1:250 | 99/101 | Hippocampus |
| GAPDH (ab9485; Abcam, UK) | Rabbit polyclonal | 1:5000 | 37 | N/A |
| Synaptophysin (MAB5258; Merck Millipore, Germany) | Mouse monoclonal | 1:1000 | 38 | Hippocampus |

**Table S3 Secondary antibodies used in immunoblotting**

| **Antibody** | **Species** | **In 5% milk + TBST** |
| --- | --- | --- |
| HRP-conjugated goat polyclonal anti-rabbit (ab6721; Abcam, UK) | Goat Polyclonal | 1:5000 |
| HRP-conjugated donkey anti-goat (ab6885; Abcam, UK) | Donkey polyclonal | 1:5000 |
| HRP-conjugated rabbit anti-mouse (A9044; Sigma-Aldrich, UK) | Rabbit | 1:40 000 |

**Table S4 Complete statistical analysis of GluK2-Neo electrophysiology and its glutamatergic modulation.**
